# Marsupial cathelicidins: characterization, antimicrobial activity and evolution in this unique mammalian lineage

**DOI:** 10.3389/fimmu.2025.1524092

**Published:** 2025-04-04

**Authors:** Emma Peel, Adele Gonsalvez, Carolyn J. Hogg, Katherine Belov

**Affiliations:** ^1^ Australian Research Council (ARC) Centre of Excellence for Innovations in Peptide and Protein Science, The University of Sydney, Sydney, NSW, Australia; ^2^ School of Life and Environmental Science, Faculty of Science, The University of Sydney, Sydney, NSW, Australia

**Keywords:** marsupial, monotreme, cathelicidin, antimicrobial, ancestral sequence reconstruction, evolution, neutrophil granule protein

## Abstract

**Introduction:**

Cathelicidins are a family of antimicrobial peptides well-known for their antimicrobial and immunomodulatory functions in eutherian mammals such as humans. However, cathelicidins in marsupials, the other major lineage of mammals, have received little attention despite lineage-specific gene expansions resulting in a large and diverse peptide repertoire.

**Methods:**

We characterized cathelicidins across the marsupial family tree and investigated genomic organisation and evolutionary relationships amongst mammals. Ancestral sequence reconstruction was used to predict ancestral marsupial cathelicidins, which, alongside extant peptides, were synthesized and screened for antimicrobial activity.

**Results:**

We identified 130 cathelicidin genes amongst 14 marsupial species representing 10 families, with gene expansions identified in all species. Cathelicidin genes were encoded in a highly syntenic region of the genome amongst all mammals, although the number of gene clusters differed amongst lineages (eutherians one, marsupials two, and monotremes three). 32 extant and ancestral marsupial cathelicidins displayed rapid, potent, and/or broad-spectrum antibacterial and antifungal activity. Phylogenetic analysis revealed that marsupial and monotreme cathelicidin repertoires may reflect both mammals and birds, as they encode non-classical cathelicidins found only in birds, as well as multiple copies of neutrophil granule protein and classic cathelicidins found only in eutherian mammals.

**Conclusion:**

This study sheds light on the evolutionary history of mammalian cathelicidins and highlights the potential of wildlife for novel bioactive peptide discovery.

## 1 Introduction

Antimicrobial peptides (AMPs) are an ancient component of innate immunity that are found across the tree of life (1). Cathelicidins are a family of small, positively charged AMPs found in vertebrates and are one of the main AMP families in mammals (2). The genes that encode cathelicidins contain four exons: exon one encodes the signal peptide, exons two and three the highly conserved cathelin domain, and exon four the highly variable antimicrobial domain (2). Cathelicidins are expressed as a prepropeptide within neutrophil granules and epithelial cells. Following secretion or de-granulation, the antimicrobial domain is proteolytically cleaved to form the active mature peptide (MP) that is approximately 20 to 50 amino acids in length (2).

Cathelicidins have pleiotropic functions, including direct antimicrobial activity, immunomodulation, wound healing, and cancer (2). Numerous studies demonstrate their broad-spectrum antimicrobial activity against bacteria, fungi, and viruses, including multi-drug-resistant strains and COVID-19 (2–6). Cathelicidins generally act via electrostatic interaction between the positively charged peptide and negatively charged microbial membrane, leading to pore formation or membrane dissolution and cell death (7). Cathelicidins also modulate the immune response; they are anti-inflammatory, are chemotactic, and influence the development of immune cells such as lymphocytes and neutrophils (8, 9). Finally, cathelicidins have diverse roles in wound healing and cancer that differ depending on the cell type and species (10, 11). Their myriad of functions and multiple mechanisms of action make cathelicidins attractive targets for therapeutic development to combat rising antimicrobial resistance (12, 13). For example, human cathelicidin LL-37 shows promise as an effective treatment for chronic leg ulcers in clinical trials (14). Iseganan, a synthetic analog of pig cathelicidin, has undergone clinical trials for the treatment of ventilator-associated pneumonia (15) and oral mucositis (16). Topical iseganan passed phase III clinical trials for the treatment of oral mucositis, significantly reducing bacterial and fungal infections in a cohort of 225 chemotherapy patients without inducing resistance (16). However, iseganan did not improve clinical outcomes in patients with ventilator-associated pneumonia, where no difference was observed between treated or placebo groups in a double-blind trial of 709 patients (15).

The number of cathelicidin genes within species varies, with only a single gene encoded by many eutherian mammals such as humans (17) and mice (18). However, recent gene duplications have occurred in some species, such as cows (19), sheep (20), and pigs (21, 22), likely in response to pathogen pressures. Cathelicidin genes are encoded in a single cluster within the genome of eutherians, which is syntenic amongst all species studied to date (19, 20, 23–25). Cathelicidins likely evolved from cysteine proteases, given the sequence similarity within the cathelin domain, hence their classification within the cystatin protein superfamily (26). The evolutionary history of vertebrate cathelicidins is unknown, however, cathelicidins in birds and mammals likely evolved from a common ancestral gene (23, 27). Some eutherians encode a single *neutrophil granule protein* gene upstream of the genomic region encoding cathelicidin(s), which also sits within the cystatin superfamily but does not undergo post-translational cleavage and is not antimicrobial (25, 28).

Eutherians are only one lineage of mammals, the others being marsupials (e.g., kangaroo) and monotremes (e.g., platypus). Marsupials diverged from eutherian mammals around 150 million years ago (MYA) (29) while monotremes diverged from therian mammals (eutherians and marsupials) around 180 MYA (30). Marsupials differ from eutherian mammals in several ways, one of which is the timing and nature of neonatal development. Marsupials have a short gestation of around 30 days, after which they give birth to altricial young that are immunologically naïve and develop within the mother’s pouch (31). Unlike the sterile *in-utero* development in eutherians, the pouch is non-sterile and contains a diverse microbiome, including potential pathogens (32–35). Numerous mechanisms have evolved to protect marsupial young during development in the pouch, including rapid development of the innate immune system and passive immunity via the milk (36). Antimicrobial peptides such as cathelicidins may also play an important role in protecting altricial young (33, 36–43). Despite this, cathelicidins have only been characterized in four marsupials: the Tasmanian devil (*Sarcophilus harrisii*) (33), koala (*Phascolarctos cinereus*) (43), tammar wallaby (*Notamacropus eugenii*) (38, 39), and gray short-tailed opossum (*Monodelphis domestica*) (44, 45). All species studied to date encode between seven and 19 cathelicidin genes that are not orthologous to those in eutherians, suggesting lineage-specific gene duplication has occurred in marsupials. Cathelicidins are expressed in marsupial milk (40, 46–48), pouch skin, and skin of the pouch young within the first few days of life (38, 39). They also have antimicrobial activity against bacteria, viruses, and fungi, including drug-resistant strains (33, 41–43, 49), some of which act synergistically with antibiotics *in vitro* (49). Given their expression profile and antimicrobial activity, marsupial cathelicidins may play an essential role in the innate immune protection of altricial young within the pouch.

Advances in genome sequencing technologies, subsequent decrease in cost, and consortia such as the Earth Biogenome Project and Vertebrate Genome Project have resulted in a substantial increase in the availability of genome assemblies for wildlife (50–53). High-quality genome assemblies and bioinformatic mining have led to the accurate annotation and evolutionary analysis of complex immune gene families in marsupials, such as the major histocompatibility complex (54–56) and defensins (57). Our aim was to characterize cathelicidins across the marsupial family tree to investigate evolution, genomic organization, and synteny amongst mammals (monotremes, marsupials, and eutherians). We characterized cathelicidins in 14 species representing 10 families: brown antechinus (*Antechinus stuartii*), fat-tailed dunnart (*Sminthopsis crassicaudata*), numbat (*Myrmecobius fasciatus*), greater bilby (*Macrotis lagotis*), eastern barred bandicoot (*Perameles gunnii*), rufous hare wallaby (hereafter mala) (*Lagorchestes hirsutus*), eastern grey kangaroo (*Macropus giganteus*), red kangaroo (*Macropus rufus*), brushtail possum (*Trichosurus vulpecula*), western ringtail possum (*Pseudocheirus peregrinus*), mahogany glider (*Petaurus gracilis*), brushtail bettong (hereafter woylie) (*Bettongia penicillata ogilbyi*), common wombat (*Vombatus urinus*) and southern hairy-nosed wombat (*Lasiorhinus latifrons*). To do this, we mined existing genomic and transcriptomic data from 11 species and generated transcriptomic data for the remaining three species. We then used ancestral sequence reconstruction (ASR) to predict ancestral marsupial cathelicidins and test their antimicrobial activity and kill kinetics against a panel of 11 bacteria and three fungi, alongside extant marsupial cathelicidins. Cathelicidins have expanded across the marsupial family tree, resulting in a unique peptide repertoire with members that have potent and rapid antimicrobial activity.

## 2 Methods

### 2.1 Blood transcriptomes

Genome assemblies were not available for the mahogany glider or southern hairy-nosed wombat, and only a highly fragmented genome was available for the rufous hare wallaby (58, 59). Similarly, transcriptomic resources were not available for these three species. As such, we generated blood transcriptomes for the mahogany glider, southern hairy-nosed wombat, and rufous hare wallaby. Thus, 500µL of whole blood was collected from a single male southern hairy-nosed wombat and a single female mahogany glider into RNAprotect animal blood tubes (Qiagen) during routine health checks. In addition, 100µL of whole blood was collected from a single female mala into RNAprotect tubes during routine health checks. All samples were stored at -80°C until extraction. Total RNA was extracted from whole blood using Qiagen RNAprotect Animal Blood Kit with on-column digestion of contaminating DNA using the RNase-free DNase I set (Qiagen). RNA was quality checked using the NanoDrop spectrophotometer, with all samples displaying A260/280 and A260/230 values of 1.86 to 2.39. RNA concentration and integrity were determined using the Agilent RNA Nano 6000 kit on the bioanalyzer, with all samples displaying an RNA integrity number (RIN) of 8.7 to 9.6. Total RNA was submitted to the Ramaciotti Centre for Genomics (The University of New South Wales) for TruSeq stranded mRNA library prep, and sequenced as 150bp paired end reads across an SP flowcell on an Illumina NovaSeq6000. This resulted in 16–23GB of raw data per sample.

### 2.2 Transcriptome assembly and annotation

Raw reads from southern hairy-nosed wombat, mahogany glider, and mala were quality checked using FastQC v0.11.8 (60), and then quality and length trimmed using Trimmomatic v0.38 (61) with the following parameters: ILLUMINACLIP:2:30:10, SLIDINGWINDOW:4:5, LEADING:5, TRAILING:5 and MINLEN:25 (61). Trimmed reads were then quality checked using FastQC v0.11.8 (60).

Trimmed reads from each species were then assembled *de novo* using Trinity v2.8.3 (62) with default parameters and without normalization. Assembly statistics were generated using TrinityStats.pl from the Trinity v2.8.3 package, and assemblies were assessed for functional completeness using BUSCO v5.2.2 with the mammalia_obd10 gene set (63) on Galaxy Australia. Trimmed reads were then mapped back to the assembly using bowtie2 v2.3.3.1 (64) to determine read representation.

Annotation was performed using Trinotate v3.1.1 (65). Briefly, TransDECODER v5.5.0 (65) was used to identify the longest open reading frame within transcripts and predict coding regions. Trinity transcripts and TransDECODER-predicted proteins were then used as input for blastx and blastp (66) searches against the Swiss-Prot non-redundant database, Tasmanian devil genome v1.11 (GCA_902635505.1) annotated proteins downloaded from Ensembl (https://asia.ensembl.org/Sarcophilus_harrisii/Info/Index), and the immunome database for marsupials and monotremes (IDMM) (67) with an e-value cutoff of 1e^-5^ and reporting only the top BLAST hit for each transcript or protein sequence. TransDECODER-predicted proteins were also used as input to HMMER v3.2 (68) to search against the Pfam database (69) to identify conserved protein domains and SignalP v4.1f (70) for predicted signal peptide regions. RNAmmer v1.2 (71) was then used to detect contamination ribosomal RNA transcripts. The output from each of these steps was then loaded into a sqlite database, and gene ontology terms assigned according to SwissProt annotations.

### 2.3 Cathelicidin gene annotation

Cathelicidin genes were characterized in the genomes and/or transcriptomes of 14 marsupial species using BLAST+ v2.7.1 (66). See **Supplementary Table 1** for details of the datasets used. Known marsupial, monotreme, and eutherian cathelicidins were used as queries, with an e-value cutoff of 10 used in all BLAST searches to ensure all potential hits were retained. Accession numbers for query sequences are available in **Supplementary Table 2**. Putative cathelicidin sequences for each species were aligned to known marsupial cathelicidins using clustalW in BioEdit (72) to confirm expected gene structure, presence of protein domains, and conserved amino acid residues and motifs. Signal sequences were identified using signalP v6.0 (73). Mature peptide sequences were predicted using the ExPasy peptide cutter (http://web.expasy.org/peptide_cutter/) with neutrophil elastase as outlined previously (33, 42, 43).

Mature peptide physiochemical properties were calculated as follows: molecular weight and charge at pH7 were calculated using Protein Calculator v3.4 (http://protcalc.sourceforge.net/). Grand average of hydropathicity (GRAVY) scores were calculated using ProtParam through the ExPasy web server (https://web.expasy.org/protparam/). Kyte and Doolittle hydropathicity plots and Deleage and Roux alpha helicity plots were both generated using ProtScale with a window size of 7 through the Expasy web server (https://web.expasy.org/protscale/). Amino acid identity and similarity were calculated based on clustalW alignments in BioEdit using the blossum62 matrix for similarity.

Cathelicidins previously identified in the Tasmanian devil (33), koala (43), tammar wallaby (38, 40), and opossum (44, 45) were annotated in the latest genome assembly of each species: GCF_902635505.1, GCA_003287225.2, GCA_028372415.1, and GCF_027887165.1, respectively (**Supplementary Table 3**). Genes flanking the cathelicidin gene cluster in the NCBI RefSeq annotation of the opossum genome (GCF_027887165.1) were used to search the genomes of 10 marsupials in this study with genomes available (**Supplementary Table 1**), as well as the latest genome assemblies of the Tasmanian devil (GCF_902635505.1), koala (GCA_003287225.2) and tammar wallaby (GCA_028372415.1) using BLAST+ v2.7.1.

### 2.4 Ancestral cathelicidin prediction

Protein sequences for full-length cathelicidins identified amongst the 14 species were aligned to published marsupial and monotreme cathelicidins from Tasmanian devil (33), koala (43), tammar wallaby (38, 40), gray short-tailed opossum (44), echidna (42), and platypus (74), as well as cathelicidins from human, mouse, cow, pig, sheep, and chicken using clustalW in BioEdit. Partial cathelicidin sequences were excluded as only full-length sequences could be input to ancestral prediction. This multiple sequence alignment was used to generate a maximum-likelihood (ML) phylogenetic tree in MEGA-X v10.2.4 using the Jones–Thornton–Taylor model and gamma distribution with four discrete categories (JTT+G4) and 200 bootstrap replicates (75). The phylogenetic tree was used as input to ANCESCON (76) and GASP (77) with default parameters to predict ancestral consensus cathelicidin sequences for each node within the tree. Where the ancestral sequence predicted by ANCESCON and GASP was not concordant, the ANCESCON sequence was selected as outlined previously (41). Ancestral sequences derived from 14 nodes within the tree were selected for synthesis based on their bootstrap support (>70%), dispersal throughout the tree, and positive charge at pH 7. Mature peptide sequences and physiochemical properties for ancestral cathelicidins were predicted using the same methods as used for extant cathelicidins.

### 2.5 Genomic organization and synteny analysis

The genomic organization of eutherian and chicken cathelicidins was investigated in the latest reference genome of each of the following species: human (GRCH38.p14, GCF_000001405.40), mouse (GRCm39, GCF_000001635.27), cow (ARS-UCD2.0, GCF_002263795.3), sheep (ARS-UI_Ramb_v3.0, GCF_016772045.2), and chicken (bGalGal1.mat.broiler.GRCg7b, GCF_016699485.2). Genes known to flank the 5’ (cell division cycle 25A - *CDC25A*) and 3’ (nucleoside diphosphate kinase 6 - *NME6*) ends of the eutherian cathelicidin cluster, and the 5’ end of the chicken cluster (Kelch-like protein 18 - *KLHL18*) (23) were identified in the publicly available genome annotation of the platypus, echidna, Tasmanian devil, antechinus, woylie, numbat, brushtail possum, wombat, and opossum (**Supplementary Table 1**). Genes not identified using this method, and for species without a genome annotation, human flanking gene proteins were downloaded from Uniprot and used as queries in a tblastn search of each genome with BLAST + v2.7.1 (66). Genomic organization was not investigated in the mahogany glider and southern hairy-nosed wombat as genomes were not available for these species.

To establish syntenic relationships within and amongst extant marsupial and eutherian cathelicidin genes, a protein multiple sequence alignment was generated in BioEdit using clustalW, which included both full-length and partial cathelicidin sequences from the same species as above, as well as rainbow trout and Atlantic salmon. Given the close relationship between chicken cathelicidins and eutherian neutrophilic granule protein (NGP), sequences from cow, mouse, and rabbit NGP were included. This alignment was used to generate a maximum-likelihood phylogenetic tree in IQ-TREE v1.6.12 (78) with ModelFinder (79) (JTT+G4) and 1000 Ultrafast bootstrap replicates (80). The resulting tree was visualized using the ggtree package in R.

### 2.6 Peptide synthesis

Predicted mature peptide sequences identified amongst 14 marsupial species were ranked according to properties known to influence antimicrobial activity such as percentage of cationic residues, charge at pH7, and amphipathicity (7, 42) and if they were a feasible length for solid phase peptide synthesis (SPPS) (less than 50 residues). From this, 64 extant and 14 ancestral cathelicidins were synthesized by ChinaPeptides Co. Ltd using SPPS and purified to greater than 95% using high-performance liquid chromatography.

### 2.7 Antimicrobial activity

**Figure 1:**
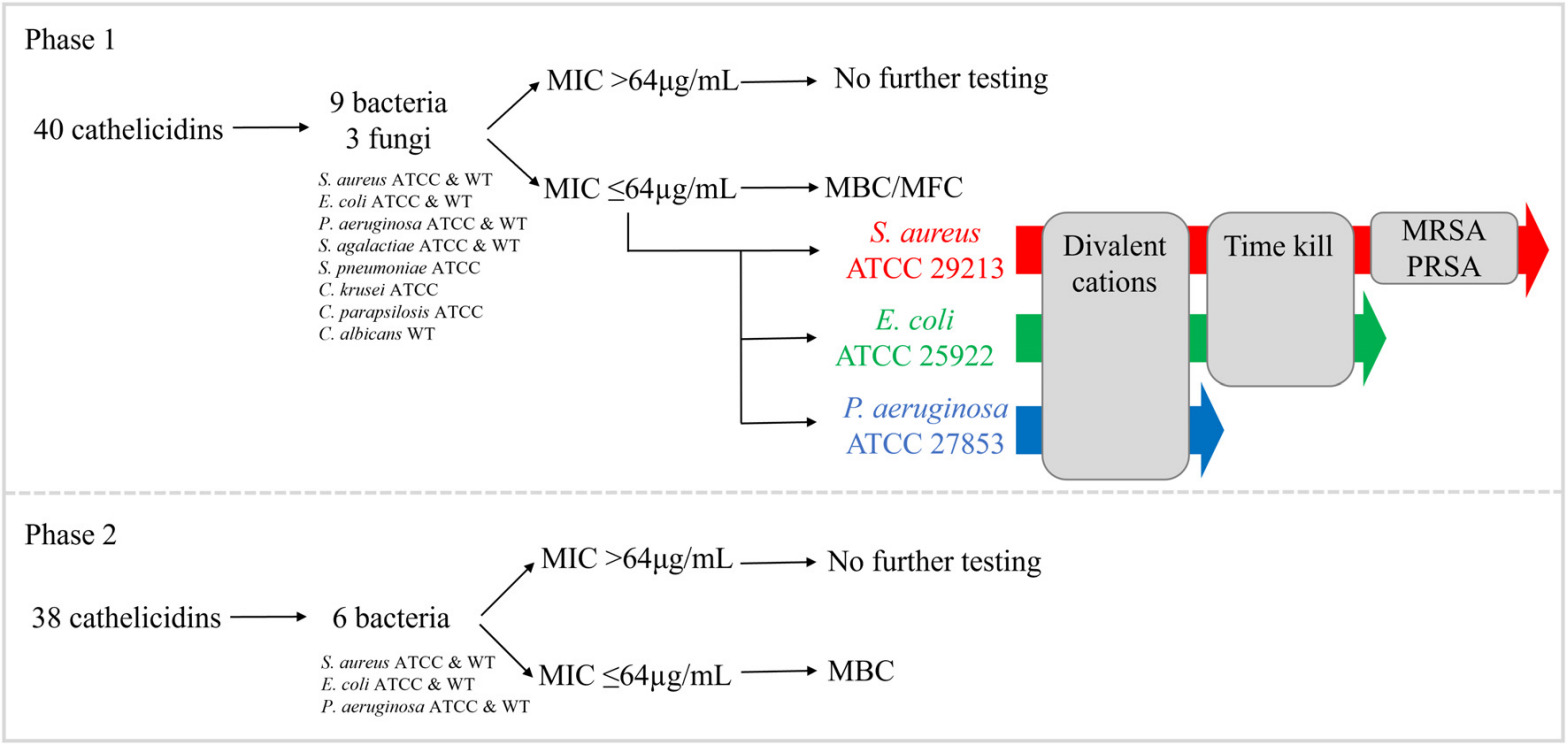
Antimicrobial testing of 78 extant and predicted ancestral marsupial cathelicidins in two phases. In phase I, 40 cathelicidins were tested against nine bacteria and three fungi. In phase II, an additional 38 cathelicidins were tested against six bacteria (same strains as phase I).

Antimicrobial testing of 78 cathelicidin MPs was performed in two phases as outlined in **Figure 1**. In phase I, 40 peptides (27 extant and 13 ancestral) were selected for testing based on rank (outlined above) and similarity to published marsupial cathelicidins with known antimicrobial activity (33, 41–43). These peptides were tested against nine bacteria (*Staphylococcus aureus* ATCC 29213 and clinical isolate, *Escherichia coli* ATCC 25922 and clinical isolate, *Pseudomonas aeruginosa* ATCC 27853 and clinical isolate, *Streptococcus agalactiae* ATCC 12386 and clinical isolate, *Streptococcus pneumoniae* ATCC 49619) and three fungi (*Candida krusei* ATCC 6258, *Candida parapsilosis* ATCC 22019, and *Candida albicans* clinical isolate). Details regarding these strains are available in **Supplementary Table 4**. The antimicrobial activity of cathelicidins was determined as the minimum inhibitory concentration (MIC), which was the lowest concentration of cathelicidin that prevented visible bacterial or fungal growth, relative to the negative control. Peptides with an MIC ≤64µg/mL underwent further tests as follows. Minimum bactericidal concentration (MBC) and/or minimum fungicidal concentration (MFC) was conducted to determine microbicidal versus static action. Activity against *S. aureus* ATCC*, E. coli* ATCC, and *Pseudomonas aeruginosa* ATCC 27853 was examined in the presence of divalent cations to determine the impact of inhibitors. Killing kinetic assays were performed for peptides with an MIC ≤64µg/mL against *S. aureus* ATCC and/or *E. coli* ATCC. Finally, peptides with an MIC ≤64µg/mL against *S. aureus* ATCC or clinical isolate were tested for activity against methicillin-resistant (MRSA) and penicillin-resistant strains (PRSA).

In phase II, the remaining 38 peptides (37 extant and one ancestral) were tested against six bacteria (*S. aureus* ATCC and clinical isolate, *E. coli* ATCC and clinical isolate, and *P. aeruginosa* ATCC and clinical isolate). Peptides with an MIC ≤64µg/mL were included in MBC tests to determine microbicidal versus static action. No further testing on phase II peptides was performed. Detailed methods for all assays are provided in subsequent sections.

Antimicrobial activity was determined using a broth microdilution susceptibility assay in 96 well polypropylene plates according to Clinical Laboratory Standards Institute guidelines M07-A and M26A as described previously (33, 42, 43). The bacterial and fungal isolates from humans and animals used in this study are outlined in **Supplementary Table 4**. Only yeasts were included in the panel of fungi as they have been used in the susceptibility testing of eutherian (3, 81–83) and other marsupial cathelicidins previously (33, 42, 43). Cathelicidin mature peptides were solubilized in DMSO and serially diluted in duplicate from 128-0.25μg/mL in a final volume of 100µL. For bacteria, cathelicidins were diluted in Mueller Hinton Broth (MHB) with or without 10% lysed horse blood, and with (MH II B) or without (MHB) calcium and magnesium cations. For fungi, cathelicidins were diluted in Roswell Park Memorial Institute medium (RPMI)-1640 MOPS. Ampicillin or tetracycline were included as a positive control for Gram-positive and -negative bacteria respectively, while amphotericin B was used for fungi. A media-only negative control and growth control (no cathelicidin) were also included on each plate. Bacteria were sub-cultured on Muller Hinton agar (MHA) or sheep blood agar (BA), and fungi on Sabouraud dextrose agar (SAB), 20–24 hours prior to the test. Colonies were suspended in saline, the concentration adjusted to a 0.5 McFarland standard, and then diluted in media to a concentration of 1.0 x 10^6^ cells/mL. Then, 100μL was added to each well of the cathelicidin dilution plate, giving a final inoculum density of 5 x 10^5^ CFU/mL and cathelicidin concentration of 64-0.125μg/mL. Colony counts were performed on MHA/BA/SAB to confirm inoculum density. All plates were incubated at 35°C for 20–24 hours, depending on the strain.

To determine the MBC for cathelicidins with an MIC ≤64μg/mL, 100μL was removed from the MIC and 2x MIC wells in duplicate, plated onto MHA or SAB, and incubated at 35°C for 20–24 hours, depending on the strain. Colonies were counted and compared to the colony count of the starting inoculum. The MBC was determined as the lowest cathelicidin concentration that resulted in a 99.9% reduction in CFU/mL, relative to the starting inoculum of 5 x 10^5^ CFU/mL. Cathelicidins that did not result in a 99.9% reduction in CFU/mL were considered bacteriostatic.

### 2.8 Killing kinetics

Killing kinetics were investigated for cathelicidins that displayed an MIC of ≤64μg/mL against *E. coli* ATCC 25922 or *S. aureus* ATCC 29213, according to the American Society of Microbiology guidelines (Time kill assay, section 5.10.2) and CLSI M26A. The killing kinetics assay was conducted using mid-logarithmic phase cultures of *E. coli* ATCC 25922 and *S. aureus* ATCC 29213 in MHB. Cultures were adjusted to a 0.5 McFarland standard, diluted in MHB, and then 100µL was added to each culture tube containing 10mL pre-warmed MHB to give a final inoculum concentration of 5 x 10^5^ CFU/mL. A sterility control of 100uL MHB, growth control (no cathelicidin), and vehicle control (DMSO) were also included. Furthermore, 10uL was immediately removed from the growth control tube, diluted 1/1000 in saline and 100µL plated onto MHA in duplicate to confirm inoculum density. To establish colony counts from 0 hours of cathelicidin treatment, 200µL was removed from each culture tube and serially diluted ten-fold in saline from 10^0^ to 10^-14^ in a total volume of 200µL. Then, 10µL of each dilution was spotted onto MHA in duplicate, which were incubated at 35°C for 24 hours.

Cathelicidin peptides were solubilized in DMSO, serially diluted two-fold in MHB, and 100uL of each peptide and dilution added to individual culture tubes to give a final cathelicidin concentration of 2x MIC to 0.125x MIC. All culture tubes were then incubated at 35°C static. At 1, 2, 4, 6, 8, 10, 12, and 24 hours post-cathelicidin treatment, 200µL was removed from each culture tube for dilution and plated on MHA as described above. The number of colonies within each 10µL spot was then counted for each dilution (100–10–14) of each cathelicidin concentration (2x MIC – 0.125x MIC) at each timepoint (0, 1, 2, 4, 6, 8, 10, 12, and 24 hours). Counts of duplicates were averaged, and the log_10_ CFU/mL graphed against time. This was used as input for one-way ANOVA (*p* < 0.05) followed by Dunnett’s *post hoc* test. To test for significant difference in bacterial growth between cathelicidins, the no-treatment control and vehicle control (DMSO solvent), the area under the curve (AUC) was calculated for each kill kinetics curve and input to a one-way ANOVA (*p* < 0.05) followed by a Dunnett’s *post hoc* test.

## 3 Results

### 3.1 Cathelicidin gene characterization

In total, 130 cathelicidin genes were characterized amongst the 14 marsupial species, with five to 15 unique genes per species (**Table 1**). The dunnart encoded the highest number of cathelicidins (n = 15), while the southern hairy-nosed wombat and mahogany glider both encoded the lowest (n = 5). Cathelicidins were expressed throughout the body in tissues of the immune, circulatory, respiratory, secretory, and endocrine systems, as well as the mammary gland and pouch skin (**Supplementary Table 3**). Cathelicidin genes were named in the order in which they were identified using the first two letters of the genus and species as per Peel et al. (33). The genomic and/or transcriptomic annotation of all cathelicidins is provided in **Supplementary Table 3**. To annotate cathelicidins in the mala, mahogany glider, and southern hairy-nosed wombat, *de novo* blood transcriptomes were assembled and annotated for each of these species. A summary of transcriptome assembly and annotation statistics is provided in the **Supplementary Material** and **Supplementary Table 5**.

**Table 1:** Number of cathelicidin genes in marsupials, monotremes, and eutherians.

Mammalian lineage	Order	Family	Species	Number of cathelicidin genes
Eutherian	Primates	Hominidae	Human	1 (17)
	Rodentia	Muridae	Mouse	1 (18)
	Artiodactyla	Bovidae	Cow	8 (19)
			Sheep	6 (20)
Marsupial	Didelmorphia	Didelphidae	Gray short-tailed opossum	19 (44, 45)
	Dasyuromorphia	Dasyuridae	Tasmanian devil	7 (33, 42)
			Brown antechinus	9^1^ (this study)
			Fat-tailed dunnart	15^6^ (this study)
		Myrmecobiidae	Numbat	9^3^ (this study)
		Thylacomyidae	Greater bilby	9^3^ (this study)
	Peramelemorphia	Peramelidae	Eastern barred bandicoot	10^6^ (this study)
	Diprotodontia	Macropodidae	Tammar wallaby	18^8^ (38, 40)
			Rufous hare wallaby	8^3^ (this study)
			Eastern grey kangaroo	13^6^ (this study)
			Red kangaroo	9^6^ (this study)
		Potoridae	Brushtail bettong	8 (this study)
		Pseudocheiridae	Western ringtail possum	7^4^ (this study)
		Petauridae	Mahogany glider	5^1^ (this study)
		Phalangeridae	Brushtail possum	14^7^ (this study)
		Vombatidae	Southern hairy-nosed wombat	5^1^ (this study)
			Common wombat	9^6^ (this study)
		Phascolarctidae	Koala	10^4^ (43)
Monotreme	Monotremata	Ornithorhynchidae	Platypus	10 (30, 74)
		Tachyglossidae	Short beaked echidna	6 (30, 42)

The number of partial genes identified is provided in superscript.

Cathelicidins previously identified in the Tasmanian devil (33), koala (43), tammar wallaby (38, 39) and opossum (44, 45) genomes were annotated in the latest genome assembly for each species. All devil and koala cathelicidins were identified. However, only 62% of tammar and 78% of opossum cathelicidins could be annotated in the latest genome assemblies (**Supplementary Table 3**). In the tammar wallaby genome, 18 cathelicidins were identified in total. This includes five previously characterized genes (*MaeuCath1, 2, 3, 4*, and *8*) and 14 new cathelicidin genes. Interestingly, *MaeuCath2* was a pseudogene in the current genome assembly, with an in-frame stop codon in exon 1. *MaeuCath4, 5* and *6* could not be identified within the latest genome assembly, even though they have previously been characterized within a mammary gland cDNA library and have been experimentally verified (38). Of the 14 new cathelicidin genes, six were characterized as full-length sequences and seven as partial sequences missing exon 2 or 4. Six of the new genes were expressed in a global transcriptome of the heart (DRX012250), spleen (DRX012251), and liver (DRX012248), including two partial sequences (*MaeuCathUNK5* and *14*) (**Supplementary Table 3**). Three of the new cathelicidin sequences identified in this study may represent sequence variants of *MaeuCath4, 5*, or *6*, as they had 56% to 77% amino acid identity to the unidentified cathelicidins. Further work is required for clarification.

Marsupial cathelicidin genes contained the characteristic sequence features of this antimicrobial peptide family. Genes were, on average, 4,699bp long and contained four exons that encoded a prepropeptide comprising three domains: a conserved signal sequence and cathelin domain and a highly variable antimicrobial domain (**Supplementary Table 3**). Full-length coding sequences were identified for 60% of the cathelicidins, while partial sequences represent the remaining 40% due to genome and/or transcriptome fragmentation. Some of these partial sequences may represent pseudogenes, although 59% of the partial sequences were expressed amongst the nine species with transcriptome data (**Supplementary Tables 1**, **3**). Additional work is required to confirm full-length coding sequences for the remaining cathelicidins.

Given that 40% of the characterized cathelicidins were partial sequences, all three peptide domains were not identified in all 130 cathelicidins. Signal sequences were identified for 116 cathelicidins, which ranged in length from 16 to 28 amino acids long and contained a high proportion of leucine residues. The cathelin domain was identified for 103 sequences, ranged in length from 81 to 100 residues, and contained three conserved motifs. The highly conserved cysteine motif (CX_10_-CX_5_-CX_16_-C) was identified for 98% of the sequences. In addition, cathelicidin motif 1 [Y-X-(ED)-X-V-X-(RQ)-A-(LIVMA)-(DQG)-X-(LIVMFY)-N-(EQ)] [Prosite PS00946 (84)] was present in 47% of the sequences, with slight modifications in a further 36%, and significant changes at the 5’ end in the remaining 17%. Cathelicidin motif 2 [F-X-(LIVM)-K-E-T-X-C-X_10_-C-X-F-(KR)-(KE)] [Prosite PS00947 (84)] was present in all 103 cathelin-domain-containing sequences.

Cathelicidin sequence composition was highly variable amongst the 130 marsupial sequences. Marsupial cathelicidins were as dissimilar to each other as they were to monotreme or eutherian cathelicidins. The average amino acid identity amongst all full-length marsupial cathelicidins identified to date (n = 107) was only 29.8%, compared to 60.9% amongst the eutherian cathelicidins used in our alignment (**Supplementary Table 4**). Marsupial cathelicidins displayed a similar level of sequence divergence to cathelicidins from monotremes (average 21.3% amino acid identity), eutherians (23.2%), and chickens (21.8%).

### 3.2 Genomic organization and synteny

**Figure 2:**
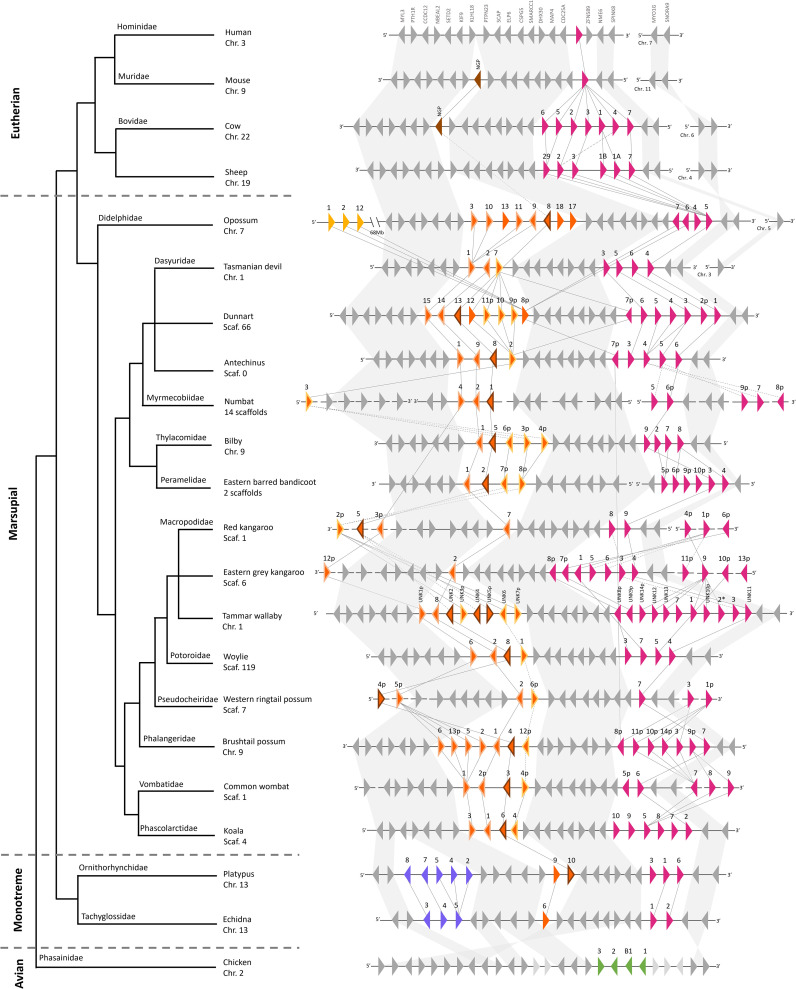
Genomic organization and synteny of cathelicidin genes within the genomes of four eutherians, 15 marsupials, two monotremes, and chicken, and a species phylogeny as per (102). Triangles represent genes: colored triangles are cathelicidin genes and gray triangles are genes that flank the cathelicidin gene clusters. The direction of the triangle represents the strand on which the gene is encoded. The number above the gene represents the cathelicidin gene name, with p indicating a partial gene sequence. * indicates a pseudogene. Cathelicidins in genomic cluster A are pink, cluster B are orange, cluster C are purple, chicken cathelicidins are green, and eutherian NGP are brown. Within cluster B, cathelicidins that are putative orthologs of *neutrophil granule protein (NGP)* are outlined in brown. Cathelicidins that cluster in the NGP-like clade are outlined in light orange. Cathelicidins that cluster in the basal clade are outlined in yellow. Genomic distances are not drawn to scale. Syntenic relationships are represented by lines: solid lines indicate a bootstrap value of greater than 95% and dashed lines indicate a bootstrap value of 90% to 95%. Bootstrap values were taken from the phylogenetic tree in **Figure 3**. Branch lengths in the species phylogeny are not accurate and are for the interpretation of species’ relationships only.

Marsupial cathelicidins were encoded in two clusters in the genome of the 15 species included in this analysis that we have labeled clusters A and B (**Figure 2**, **Supplementary Table 3**). Genome organization could not be investigated in the mahogany glider and southern hairy-nosed wombat as genomes are not available for these species and in mala as the genome is highly fragmented. Monotreme cathelicidins were encoded in three clusters in the genome of the two species studied: clusters A, B and C. Using the latest high-quality genome assemblies, we were able to resolve the genomic organization of cathelicidins previously characterized in the devil, koala, tammar wallaby, opossum, platypus, and echidna (**Supplementary Table 6**).

**Figure 3:**
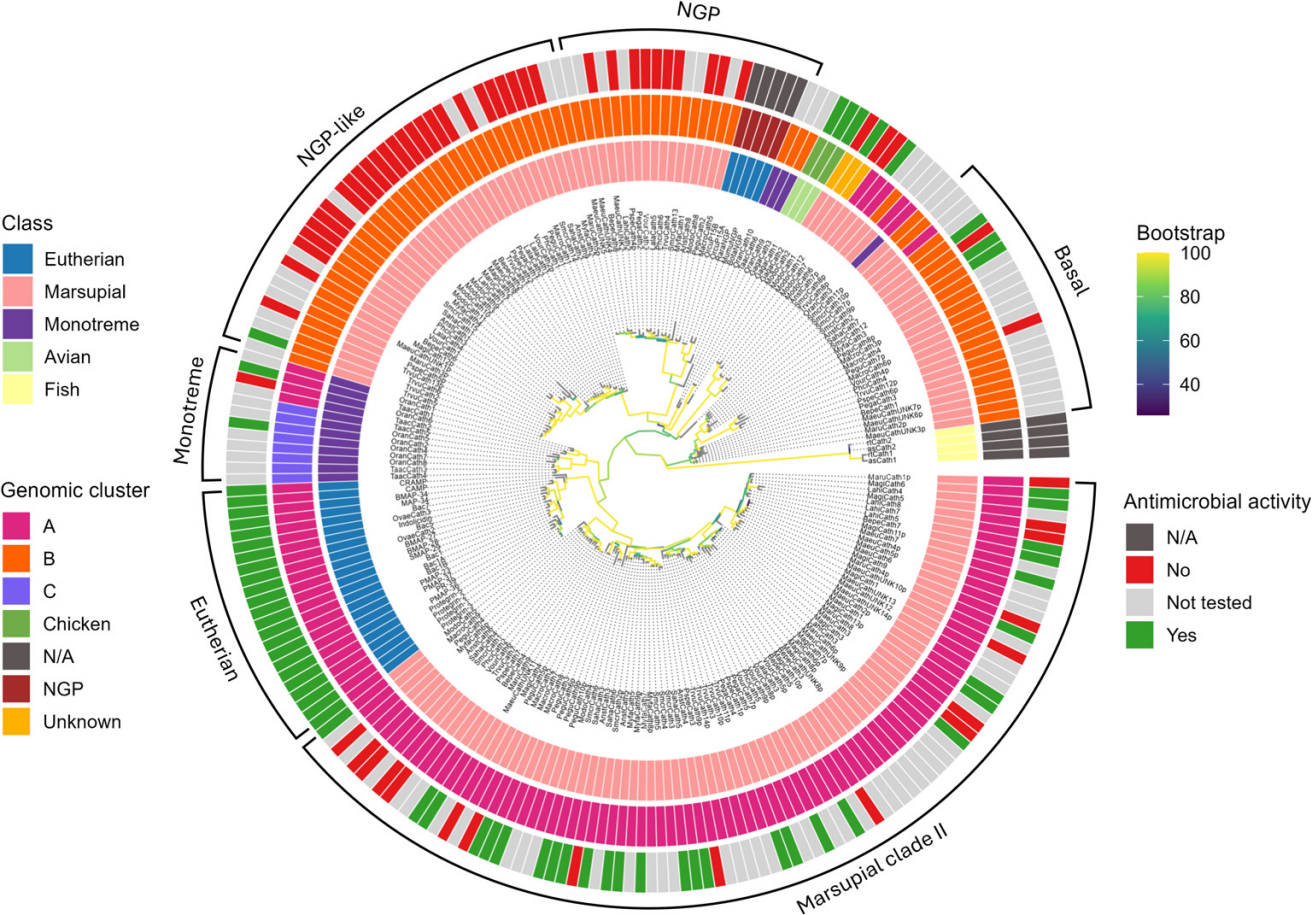
Phylogenetic relationships amongst marsupial, monotreme, eutherian, and chicken cathelicidins, and eutherian neutrophil granule proteins. The maximum likelihood tree is rooted with fish cathelicidins. The branches are colored according to bootstrap support. The major clades are marked marsupial clade I (consisting of basal, NGP, and NGP-like clades) and marsupial clade II. The monotreme-specific and eutherian-specific clades at the base of marsupial clade II are shown for comparison. The three colored circles surrounding the tree are as follows. The inner circle is colored according to class, the middle circle according to genomic cluster (**Figure 2**), and the outer circle according to antimicrobial activity (**Tables 3**, **4**). Accession numbers for sequences within this tree are provided in **Supplementary Table 2**.

Cathelicidin gene cluster A was present in the genomes of all mammals studied (eutherian, marsupial and monotreme), as shown by the pink triangles in **Figure 2**. Cluster B was encoded upstream of A and was only found in the genomes of marsupials and monotremes, as shown by the orange triangles in **Figure 2**. Clusters A and B encoded a similar number of genes in the marsupial and monotreme species studied, with an average size of 121 and 143kb, respectively (**Supplementary Tables 3**, **7**). Cluster C was encoded upstream of B and was specific to monotremes, as shown by the purple triangles in **Figure 2**. A third cathelicidin gene cluster was identified in the opossum genome, 68Mb upstream of cluster B, and flanked by long non-coding RNA at both ends (**Figure 2**). Phylogenetic evidence indicates these genes likely originated from cluster A (**Figure 3**). A comparison of cluster gene number and size is provided in **Supplementary Table 7**.

Clusters A and B were encoded on the same chromosome/scaffold in high-quality marsupial genomes (opossum, devil, koala, tammar wallaby, Tasmanian devil, woylie, antechinus, brushtail possum, bilby, and dunnart) within a 1.2 to 1.6Mb region amongst these ten species (**Supplementary Table 3**). Similarly, clusters A, B and C were encoded on the same chromosome in the high-quality platypus and echidna genome within a 0.7Mb and 1.3Mb region respectively (**Supplementary Table 3**). We were unable to completely resolve the genomic organization of clusters A and B in draft-quality marsupial genomes (red kangaroo, eastern grey kangaroo, western ringtail possum, rufous hare wallaby, common wombat, and numbat), as multiple orphan cathelicidin genes were located on individual short scaffolds or clusters A and B were located on different scaffolds (eastern barred bandicoot) (**Figure 2**). Phylogenetic evidence was used to assign orphan genes to clusters for these species. Genomic organization could not be investigated in the mala as the cathelicidin genes were highly fragmented, with exons encoded on individual short scaffolds (**Supplementary Table 3**).

Cathelicidin gene clusters were encoded within a highly syntenic region of the genome amongst the eutherians, marsupials and monotremes studied (**Figure 2**). *CDC25A* flanked the 5’ end of cluster A in all the marsupials and monotremes studied, as in the eutherians (**Figure 2**; **Supplementary Table 3**). The genomic region upstream of *CDC25A* in eutherians was syntenic with the region upstream of cluster A in marsupials and monotremes, with highly conserved gene order and orientation in most species (**Figure 2**; **Supplementary Table 3**). This includes *KLHL18*, which flanked the 5’ end of cluster B in marsupials and monotremes (**Figure 2**), and *PTH1R*, which flanked the 5’ end of cluster C in monotremes.

### 3.3 Phylogeny

Marsupial cathelicidins formed two large clades within the phylogenetic tree that we have labeled clades I and II (**Figure 3**). All mammalian cathelicidins within the tree generally grouped according to genomic cluster (A, B, or C). Cathelicidins in clade I generally formed three orthologous clusters (**Figure 3**). One orthologous cluster contained genes from 16 marsupials, eutherian *neutrophilic granule protein (NGP)* and one platypus cathelicidin, with 100% bootstrap support for this clade. These cathelicidins were encoded in the middle of cluster B in the genome of all marsupials and platypus (**Figure 2**; **Supplementary Table 3**). Cathelicidins from eastern grey kangaroo, Tasmanian devil, and echidna did not cluster within this group. This cluster has been labeled NGP in **Figure 3B**.

The second orthologous cluster within clade I contained only marsupial cathelicidins (100% bootstrap) and lies sister to the NGP cluster, with 94% bootstrap support for this topology (**Figure 3**). This cluster contained 38 cathelicidins from all 18 marsupials studied to date, and included marsupial orthologs and species-specific duplications in opossum, brushtail possum, and southern hairy-nosed wombat (**Figure 3**). These genes were encoded at the 5’ end of cluster B in the genome all species, flanked by *KLHL18* (**Figure 2**; **Supplementary Table 3**). As bootstrap support was insufficient for a direct orthologous relationship between this cluster and *NGP*, this cluster has been labeled as NGP-like in **Figure 3**. The NGP and NGP-like clusters are bound by chicken and monotreme cathelicidins (*OranCath9* and *TaacCath6*), with 77% bootstrap support for this relationship (**Figure 3**).

The third orthologous cluster lies at the base of clade I and contained orthologs from 14 marsupial species with 83% bootstrap support for this clade, labeled basal in **Figure 3**. All cathelicidins within this cluster were encoded at the 3’ end of cluster B within the genome of all species except the tammar wallaby (**Figure 2**; **Supplementary Table 3**).

Marsupial cathelicidins form a second large clade within the phylogenetic tree, labeled clade II in **Figure 3**. This clade is sister to eutherian cathelicidins (90% bootstrap) and bound by a monotreme-specific cluster (89% bootstrap). Marsupial and monotreme cathelicidins in clade II are not orthologous to those in eutherians but likely share a common ancestor. Clade II contained a marsupial-specific ortholog, with representative sequences from 15 of 18 species studied to date, with 100% bootstrap support for this clade. The remaining marsupial cathelicidins in clade II cluster according to scientific order: Peramelemorphia, Didelmorphia, Diprotodontia, and Dasyuromorphia (**Figure 3**). All marsupial cathelicidins in clade II were encoded in genomic cluster A within the genome of all species (**Figure 2**; **Supplementary Table 3**).

### 3.4 Mature peptide characterization

In total, 83 cathelicidin MP sequences were predicted from 130 cathelicidin genes amongst the 14 species (**Supplementary Table 3**). Predicted MP were highly variable in terms of length, physiochemical properties, and sequence composition, both within and amongst species. All physiochemical properties of predicted MPs are outlined in **Supplementary Table 3**. MPs shared on average 9.3% amino acid identity amongst all 111 characterized marsupial MP to date (83 from this study and 28 published), and from 4.3% (wombat) to 14.3% (dunnart) amongst MPs within an individual species.

Predicted MPs ranged in length from 10 to 50 amino acids with a molecular weight of 1038 to 5600g/mol. Over 93% of MP were positively charged at pH 7, with a range of -5.1 to +13.9 and an average of +6.6 (**Supplementary Table 3**). More than 87% of MP were hydrophilic based on negative GRAVY scores (**Supplementary Table 3**), which is also reflected in the low proportion of cationic residues (26.4% average) and average Kyte and Doolittle hydropathicity plot (**Supplementary Figure 1**). Despite this, 81% of MP were amphipathic according to Kyte and Doolittle hydropathicity plots. There was no pattern to the location of hydrophobic regions amongst the 83 peptides, hence, the average hydropathicity scores were negative (**Supplementary Figure 1**).

The majority of marsupial MP are likely alpha helical, as 81 MP contained alpha helical regions according to the Deleage and Roux alpha helicity plots where scores rose above the 0.99 threshold (**Supplementary Figure 1**). There was no pattern to the location of alpha helices amongst all 81 peptides, with helical regions at both the N- and/or C-terminus. The remaining two MPs (MyfaCath3 and AnstCath2) that were not alpha helical likely exist as linear peptides, as only the first three scores in the Deleage and Roux plot rose above the 0.99 threshold. MyfaCath3 and AnstCath2 were both rich in proline (33%) and arginine (30%), and organized in Pro-Arg-Pro repeats (85). SmcrCath12 was also rich in arginine (32%), and 11 MPs were rich in lysine (≥20%) (**Supplementary Table 3**). Seven of the MPs that were predicted to be alpha helical also contained paired cysteine residues that may form a disulfide bond. An additional MP (LahiCath8) contained an unpaired cysteine that may form homodimers through intermolecular disulfide bond formation (86). Of the 83 MPs, 64 were selected for synthesis (**Supplementary Table 3**).

### 3.5 Ancestral prediction

In total, 14 predicted ancestral cathelicidin sequences were selected from ANCESCON and GASP analysis and named marsupial ancestral peptide (MAP) 1 to 14 (**Supplementary Table 3**). The phylogenetic tree used for prediction is provided in **Supplementary Figure 2**, including the annotation of nodes within the tree that correspond to the 14 predicted ancestral sequences. The predicted MPs for each of the 14 MAP sequences ranged in length from 23 to 38 amino acids, with an average charge at pH7 of +9 (**Supplementary Table 3**). All predicted MAP MPs are likely hydrophilic based on negative GRAVY scores, which is also reflected in the Kyte and Doolittle hydropathicity plot (**Supplementary Figure 3**). All predicted MPs contained alpha helical regions, except MAP3, which is likely linear. MAP3 was also the only predicted ancestral peptide rich in arginine (32.2%) and proline (25.8%). Seven other MAPs were rich in lysine, containing over 20% lysine residues. Only one MAP (MAP10) contained paired cysteine residues that may form a disulfide bond (**Supplementary Table 3**). Predicted MPs from all 14 ancestral cathelicidins were synthesized (MAP1 to MAP14, **Supplementary Table 3**).

### 3.6 Antimicrobial activity

In total, 32 of the 78 marsupial predicted MPs tested displayed antimicrobial activity, interpreted as an MIC of equal to or less than 64µg/mL against the strains tested. The 32 MPs consist of 24 peptides from extant marsupials and 8 predicted ancestral peptides. A summary of antimicrobial activity is provided in **Table 2** and detailed in **Supplementary Table 8**. Of the MPs screened in phase I against bacteria and fungi, 68% displayed antimicrobial activity: 12 were only active against bacteria, 1 was only active against fungi, and 10 were active against both. Of the MPs screened in phase II only against bacteria, 23% were active against at least one bacteria tested.

**Table 2:** Antimicrobial activity of 78 predicted marsupial MP screened in two phases (I and II, refer to **Figure 1**).

	Screening phase	
	Phase I	Phase II
No. MP tested	40	38
No. active MP	23^8^	9^0^
Antibacterial & antifungal	10^4^	N/A
Antibacterial only	12^3^	9^0^*
Antifungal only	1^1^	N/A*

The number of predicted ancestral peptides within each category is displayed in superscript. *MPs screened in phase II were not tested for antifungal activity, hence, it is unknown if active MPs were only active against bacteria.

#### 3.6.1 Antibacterial activity

In total, 31 cathelicidins had antibacterial activity: 24 from extant marsupials and seven predicted ancestral peptides. MIC values ranged from 4 to 64µg/mL (**Table 3**). Moreover, 96% were active against the Gram-negative bacteria *E. coli* and/or *P. aeruginosa* (n = 30), 45% were active against the Gram-positive bacteria *S. aureus* (n = 14), and 41% were active against both Gram-negative and Gram-positive bacteria (n = 13) (**Table 3**). Four cathelicidins were active against all six bacteria screened in phases I and II: one from the mahogany glider (PegaCath4) and three ancestral cathelicidins (MAP8, 10, and 14) (highlighted gray within **Table 3**). Extant and ancestral marsupial cathelicidins were also active against MRSA and PRSA; 91% of the MPs tested against these strains had an MIC of 8 to 64µg/mL (**Table 3**). Overall, predicted ancestral cathelicidins displayed more broad-spectrum activity than their extant relatives. Furthermore, 85% of ancestral cathelicidins were active against at least two bacterial genera, compared to only 41% amongst extant cathelicidins (**Table 3**). The most potent cathelicidins were numbat MyfaCath7, mahogany glider PegaCath4, and western ringtail possum PspeCath1, all with an MIC of 4 µg/mL against at least one strain (highlighted gray within **Table 3**). These cathelicidins were up to four times more potent than the antibiotic tetracycline against *P. aeruginosa*, and displayed an equivalent MIC to ampicillin against *E. coli*, but not other Gram-positive bacteria (**Table 3**). Generally, the other cathelicidins with antibacterial activity had MICs higher than ampicillin and tetracycline (**Table 3**). Most cathelicidins were bactericidal rather than bacteriostatic, with an MBC ranging from 4 to 64µg/mL amongst the 31 peptides. However, the MBC was typically two-fold higher than the MIC (**Table 3**).

**Table 3:** Minimum inhibitory concentration (100) (MIC) of extant and predicted ancestral marsupial cathelicidin mature peptides against two Gram-positive and seven Gram-negative bacteria.

Species	Mature peptide	*S. aureus* ATCC	*S. aureus*	*E. coli* ATCC	*E. coli*	*P. aeruginosa* ATCC	*P. aeruginosa*	*S. agalactiae* ATCC*	S. agalactiae*	*S. pneumoniae* ATCC*	MRSA^+^	PRSA^+^
Antechinus	AnstCath2	>64	>64	16 (16)	32 (64)	>64	>64	N/A	N/A	N/A	N/A	N/A
	AnstCath4	32 (32)^32^	32 (32)^32^	64 (64)^64^	32 (64)^64^	>64	>64	>64	>64	>64	32 (64)	16 (16)
	AnstCath5	16 (32)^16^	8 (16)^8^	16 (32)^16^	32 (64)^32^	>64	32 (64)^64^	>64	32 (32)	>64	16 (32)	16 (16)
Fat-tailed dunnart	SmcrCath3	16 (16)^16^	16 (16)^16^	16 (16)^32^	8 (16)^16^	>64	64 (>64)^>64^	>64	>64	>64	16 (16)	8 (16)
	SmcrCath6	>64	>64	32 (64)^64^	>64	>64	>64	>64	>64	>64	N/A	N/A
	SmcrCath12	>64	>64	8 (8)	16 (16)	>64	>64	N/A	N/A	N/A	N/A	N/A
Numbat	MyfaCath3	>64	>64	8 (8)	16 (16)	>64	>64	N/A	N/A	N/A	N/A	N/A
	MyfaCath5	32 (64)^16^	16 (32)^16^	32 (64)^32^	32 (32)^32^	>64	>64	>64	>64	>64	32 (32)	8 (8)
	MyfaCath7	8 (8)^8^	8 (8)^8^	4 (4)^8^	4 (4)^4^	>64	32 (>64)^64^	>64	32 (32)	>64	8 (8)	4 (4)
Eastern #bababa kangaroo	MagiCath3	>64	>64	64 (>64)	>64	>64	>64	N/A	N/A	N/A	N/A	N/A
	MagiCath6	64 (64)	64 (64)	32 (32)	64 (64)	>64	>64	N/A	N/A	N/A	N/A	N/A
Rufous hare wallaby	LalaCath3	8 (16)^8^	8 (16)^8^	32 (32)^32^	64 (64)^>64^	>64	>64	>64	32 (32)	>64	8 (16)	4 (8)
Red kangaroo	MaruCath4	>64	>64	8 (8)^8^	32 (32)^32^	>64	>64	>64	>64	>64	N/A	N/A
	MaruCath9	32 (32)	32 (32)	>64	>64	>64	>64	N/A	N/A	N/A	N/A	N/A
Eastern barred bandicoot	PeguCath3	>64	>64	64 (64)	>64	>64	>64	N/A	N/A	N/A	N/A	N/A
Mahogany glider	PegaCath4	32 (>64)^64^	64 (>64)^64^	4 (8)^4^	4 (4)^2^	8 (16)^8^	8 (16)^8^	>64	>64	>64	64 (>64)	16 (>64)
Brushtail possum	TrvuCath2	>64	>64	64 (64)^64^	64 (64)^64^	>64	>64	>64	>64	>64	N/A	N/A
Woylie	BepeCath4	32 (64)	32 (32)	16 (16)	16 (32)	>64	>64	N/A	N/A	N/A	N/A	N/A
	BepeCath7	>64	>64	32 (64)^32^	32 (64)^32^	>64	>64	>64	>64	>64	N/A	N/A
Western ringtail possum	PspeCath1	>64	>64	4 (8)^4^	4 (4)^2^	8 (8)^8^	8 (16)^8^	>64	>64	>64	N/A	N/A
Bilby	MacroCath7	>64	>64	32 (64)^32^	64 (64)^64^	>64	>64	>64	>64	>64	N/A	N/A
	MacroCath8	>64	>64	>64	64 (>64)^64^	>64	>64	>64	>64	>64	N/A	N/A
Southern hairy-nosed wombat	LahiCath4	>64	>64	32 (64)^64^	16 (64)^64^	>64	>64	>64	>64	>64	N/A	N/A
	LahiCath5	>64	>64	16 (16)	16 (32)	>64	>64	N/A	N/A	N/A	N/A	N/A
Predicted ancestral cathelicidin	MAP6	>64	>64	32 (32)^32^	32 (32)^32^	>64	>64	>64	>64	>64	N/A	N/A
	MAP7	>64	>64	8 (16)^8^	16 (16)^16^	32 (32)^32^	32 (64)^32^	64 (64)	64 (64)	32 (32)	N/A	N/A
	MAP8	32 (32)^32^	32 (32)^32^	16 (32)^16^	16 (16)^8^	16 (16)^16^	16 (32)^16^	64 (64)	32 (32)	32 (32)	64 (>64)	16 (32)
	MAP10	16 (32)^16^	16 (16)^16^	8 (16)^8^	8 (8)^8^	16 (16)^16^	16 (32)^16^	64 (64)	64 (64)	64 (>64)	16 (32)	8 (16)
	MAP11	16 (32)^32^	32 (32)^16^	32 (64)^32^	32 (32)^64^	>64	>64	>64	>64	>64	32 (32)	16 (16)
	MAP14	32 (32)^32^	32 (32)^32^	8 (8)^8^	8 (8)^4^	16 (16)^8^	16 (16)^16^	64 (64)	32 (32)	32 (32)	64 (64)	16 (16)
	MAP14	32 (32)^32^	32 (32)^32^	8 (8)^8^	8 (8)^4^	16 (16)^8^	16 (16)^16^	64 (64)	32 (32)	32 (32)	64 (64)	16 (16)
Controls	Ampicillin	1^2^	0.25^0.25^	4^8^	>64	N/A	N/A	0.25	0.25	>64	N/A	N/A
	Tetracycline	N/A	N/A	N/A	N/A	32^32^	32^32^	N/A	N/A	N/A	N/A	N/A

Only peptides with an MIC >64µg/mL against at least one strain are shown. The minimum bactericidal concentration (MBC) is displayed in brackets. The MIC in the presence of cations (cation-adjusted Mueller Hinton broth) is displayed in superscript. The most potent and/or broad-spectrum peptides tested are highlighted in gray. *Only peptides from phase I were tested against *Streptococcus* species. ^+^Only peptides from phase I that had an MIC less than or equal to 64µg/mL against *Staphylococcus aureus* were tested against methicillin- (MRSA) and penicillin-resistant (MRSA) strains. N/A indicates the peptide was not tested against these strains. For µM conversion of the table below, see **Supplementary Table 8**.

Divalent cations did not impact the antibacterial activity of most cathelicidins against most of the six strains tested. This was particularly the case for *E. coli*, where 72% and 50% of peptides active against the ATCC and clinical strain, respectively, were not impacted by the presence of divalent cations. This is compared to 36%/45% of cathelicidins active against *S. aureus* and 22%/31% against *P. aeruginosa* for ATCC and clinical isolates, respectively (**Table 3**). The activity of some cathelicidins was attenuated, with a two-fold or four-fold increase in MIC, or complete attenuation (MIC >64µg/mL) depending on the strain. Interestingly, there were seven occurrences where divalent cations decreased the MIC two-fold. Of the four peptides with antibacterial activity against all six strains (PegaCath4, MAP7, 9 and 13), only the three ancestral peptides showed no attenuation in the presence of divalent cations for all six strains (**Table 3**).

#### 3.6.2 Killing kinetics

**Figure 4:**
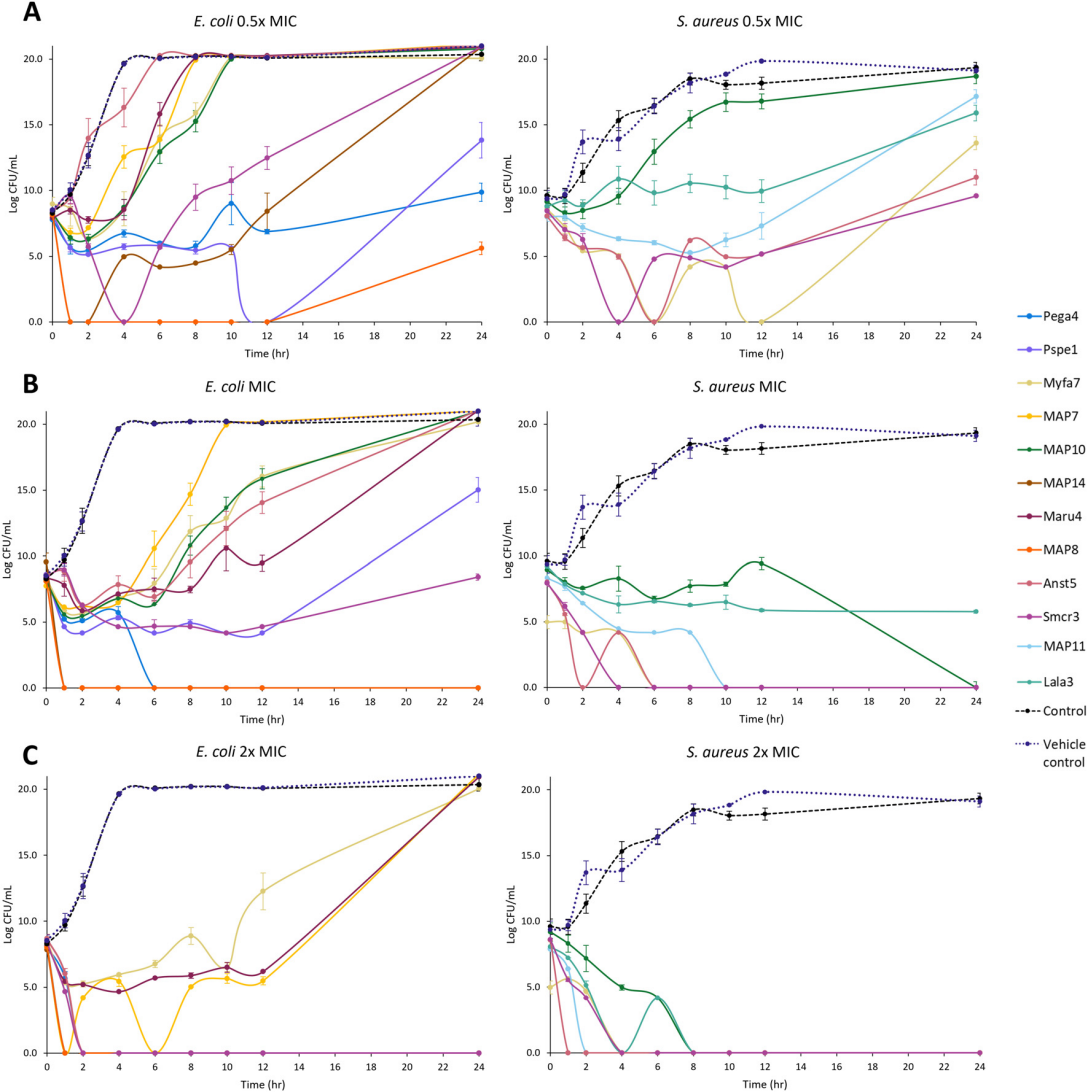
Time kill kinetics curves of 10 cathelicidins against *E*. *coli* ATCC and six cathelicidins against *S. aureus* ATCC. Results are shown for peptides tested at half the concentration of the MIC **(A)**, the MIC **(B)**, and two-fold higher concentration than the MIC **(C)**. For MIC values, see **Table 3**. *n* = 2; values are mean ± standard error of the mean (SEM).

**Figure 5:**
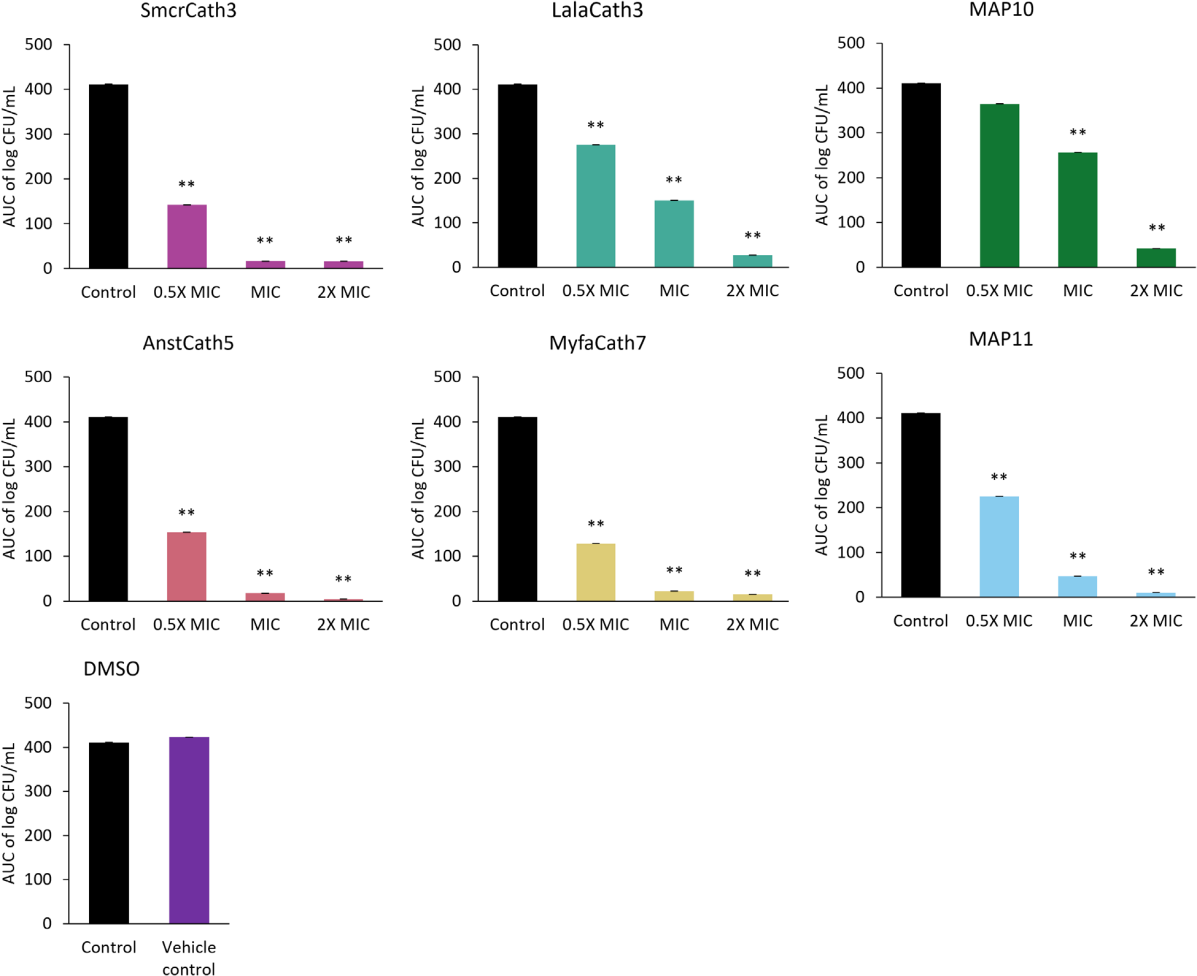
AUC of *S. aureus* ATCC time kill kinetics curve for six cathelicidins tested at three concentrations: half the MIC (0.5x MIC), the MIC, and two-fold higher than the MIC (2x MIC). The DMSO vehicle control is also shown. For MIC values, see **Table 3**. *n* = 2; values are mean ± SEM. **p < 0.05 compared to the control (one-way ANOVA followed by Dunnett’s *post hoc* test).

**Figure 6:**
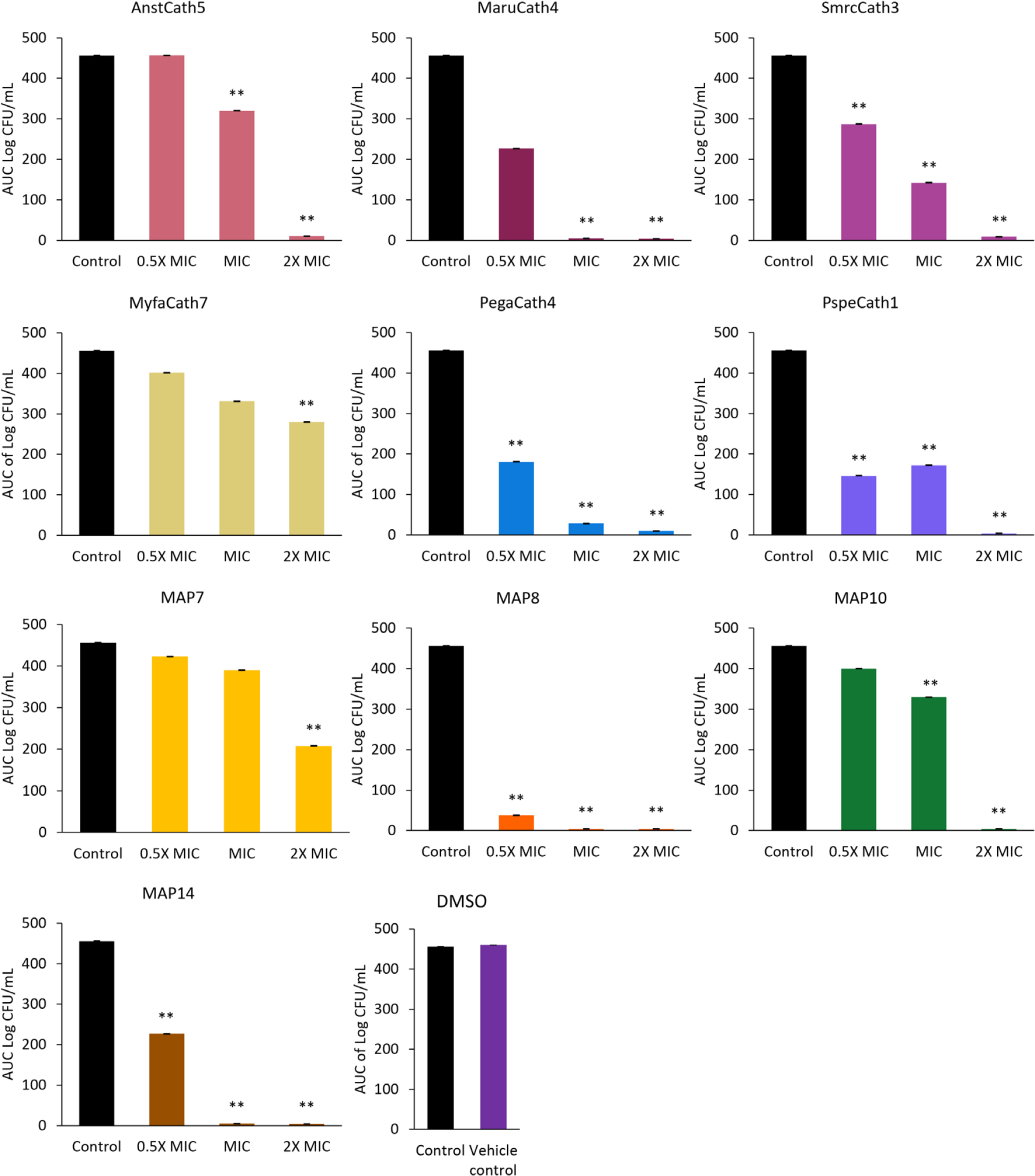
AUC of *E. coli* ATCC time kill kinetics curve for 10 cathelicidins tested at three concentrations: half the MIC (0.5x MIC), the MIC, and two-fold higher than the MIC (2x MIC). The DMSO vehicle control is also shown. For MIC values, see **Table 3**. *n* = 2; values are mean ± SEM. **p < 0.05 compared to the control (one-way ANOVA followed by Dunnett’s *post hoc* test).

Killing kinetics were investigated for six cathelicidins against *S. aureus* ATCC (Myfa7, Lala3, Anst5, Smcr3, MAP9, and MAP10) and 10 cathelicidins against *E. coli* ATCC (Myfa7, Anst5, Smcr3, Pspe1, Pega4, Maru4, and MAP6, 7, 9, and 13) (**Figure 4**). Cathelicidins rapidly knocked down bacterial growth within 4 hours of treatment (**Supplementary Tables 9**, **10**). This killing effect was sustained for 24 hours for some cathelicidins (**Figure 4**), as shown by a significant reduction in AUC compared to the no-treatment control (**Figures 5**, **6**). For others, bacterial growth recovered to the level of the no-treatment control within 24 hours (**Figure 4**). Of the four cathelicidins tested against *S. aureus* and *E. coli* (AnstCath5, MyfaCath7, SmcrCath3, and MAP10), SmcrCath3 displayed the most broad-spectrum and potent kill kinetics as it significantly reduced the growth of both bacteria (*p* < 0.05) at all three concentrations tested (0.5x MIC, MIC, and 2x MIC) (**Figures 5**, **6**).

All six cathelicidins tested against *S. aureus* caused a significant reduction in bacterial growth at the MIC relative to the no-treatment control, as shown by the AUC in **Figure 5** (*p* < 0.05). This effect was observed within 2 to 4 hours of treatment, depending on the cathelicidin (**Figure 4**; **Supplementary Table 9**). At half the MIC, all cathelicidins except MAP10 retained this activity (*p* < 0.05) with rapid knockdown within 2 to 4 hours of treatment (**Figure 4**; **Supplementary Table 9**). As expected, cathelicidins displayed more potent and rapid kill kinetics at 2x the MIC, with a greater reduction in AUC (**Figure 5**) and knockdown within 2 hours for all cathelicidins but MAP10 (**Supplementary Table 9**).

Eight out of 10 cathelicidins tested against *E. coli* ATCC caused a significant reduction in bacterial growth at the MIC relative to the no-treatment control (*p* < 0.05), as shown by the AUC in **Figure 6**. This effect was observed within 1 to 6 hours of treatment, depending on the cathelicidin (**Figure 4**; **Supplementary Table 10**). Killing kinetics were attenuated at half the MIC, as only five cathelicidins (SmcrCath3, PegaCath4, PspeCath1, MAP8, and MAP14) significantly reduced *E. coli* growth relative to the no-treatment control (**Figure 6**) within 1 to 6 hours of treatment (**Figure 4**, **Supplementary Table 10**) (*p* < 0.05). An additional three cathelicidins (MaruCath4, MyfaCath7, and MAP10) caused a significant reduction in bacterial growth within 4 hours of treatment (*p* < 0.05) (**Figure 4**; **Supplementary Table 9**). However, growth recovered to the level of the no-treatment control within 24 hours (**Figure 4**), hence the AUC was not significant (**Figure 5**). All 10 cathelicidins caused a significant reduction in *E. coli* growth at 2x the MIC (*p* < 0.05) (**Figures 4**, **6**).

#### 3.6.3 Antifungal activity

In total, 11 cathelicidins displayed antifungal activity with an MIC ≤ 64µg/mL against at least one of the three species tested (**Table 4**). Six cathelicidins were from extant marsupials, and five predicted ancestral peptides. All 11 cathelicidins were active against *C. parapsilosis*, 72% were active against *C. krusei*, and 27% were active against *C. albicans*. Antechinus AnstCath4 and AnstCath4, numbat MyfaCath7, and dunnart SmcrCath3 had antifungal activity against all three species. All 11 cathelicidins were fungicidal against at least one species, either at the MIC or two-fold higher concentration. Fungistatic activity was also observed (**Table 4**).

**Table 4:** Minimum inhibitory concentration (100) (MIC) of extant and predicted ancestral marsupial cathelicidin MP against three fungi.

Species	Mature peptide	*C. parapsilosis* ATCC	*C. krusei* ATCC	*C. albicans*
Antechinus	AnstCath4	64 (>64)	32 (64)	64 (64)
	AnstCath5	32 (>64)	32 (32)	64 (>64)
Fat-tailed dunnart	SmcrCath3	32 (32)	16 (32)	32 (64)
Numbat	MyfaCath5	64 (64)	16 (16)	>64
	MyfaCath7	32 (32)	16 (16)	64 (64)
Western ringtail possum	PspeCath1	32 (>64)	>64	>64
Predicted ancestral peptide	MAP8	32 (64)	64 (>64)	>64
	MAP9	64 (>64)	32 (64)	>64
	MAP10	32 (>64)	>64	>64
	MAP13	32 (>64)	>64	>64
	MAP14	16 (32)	64 (>64)	>64
Control	Amphotericin B	0.5	1	0.5

In total, 40 peptides were tested and only peptides with an MIC >64µg/mL against at least one species are shown. The minimum fungicidal concentration (MFC) is displayed in brackets. MPs that were active against all three strains are highlighted in gray.

## 4 Discussion

We characterized 130 cathelicidins in 14 marsupial species from 10 of 14 extant families, some of which were antimicrobial and had rapid and potent kill kinetics. Cathelicidins were encoded in a highly syntenic region of the genome across monotremes, marsupials, and eutherians, which also shared similarity to the region encoding chicken cathelicidins (23). Marsupials and monotremes encode cathelicidins that are closely related to those in eutherians and share similar physiochemical and antimicrobial properties. However, they also encode additional cathelicidins that are more similar to chicken cathelicidins and were generally not antimicrobial in our assays. In addition, we have identified multiple orthologs of neutrophil granule protein in all 18 marsupials in this study, a protein within the cystatin superfamily (as are cathelicidins) that has been found in some eutherians but lost in humans and other primates (25). Altogether, marsupials and monotremes exhibit aspects of both bird and mammalian cathelicidin gene repertoires, which suggest they have retained part of the amniote ancestral state that may have been lost in eutherian mammals.

Marsupials have a large and complex repertoire of cathelicidins and cathelicidin-like peptides, which may be linked to their unique reproduction, development, and immunology. Multiple cathelicidin genes have been identified in all marsupial and monotreme species studied to date, representing most extant families (30, 33, 38, 39, 42–44, 74). As such, the cathelicidin gene family has likely expanded in all marsupials through gene duplication, given their genomic organization and evolutionary relationships. Some marsupial cathelicidins are likely ancient and have been conserved throughout evolution, such as the marsupial-specific orthologs in clade II in the phylogenetic tree (**Figure 2**). More recent duplication events have also occurred after the divergence of marsupial families, as evidenced by the arrangement of genes according to family in clade II (**Figure 2**). In comparison, only a single cathelicidin gene is found in human (17), mouse (18), and many other eutherian mammals (2). The large marsupial cathelicidin repertoire is widely expressed in multiple tissues, including in mammary gland and pouch skin. Cathelicidins are expressed within 5 days of birth in the tammar wallaby (38, 39) and are found in Tasmanian devil (46) and koala milk (47). As such, the need for additional immunological protection during early life may have encouraged the expansion of cathelicidins in marsupials and monotremes.

### 4.1 Classic cathelicidins with antimicrobial functions

Many marsupial and monotreme cathelicidins were closely related to the classic cathelicidins found in eutherians. These cathelicidins sit within clade II in the phylogenetic tree, sister to eutherians but were not orthologous (**Figure 3**). Classic marsupial cathelicidins were encoded in cluster A in the genome of all marsupials and monotremes studied, syntenic to the single cathelicidin gene/cluster in eutherians (**Figure 2**). Many of these were antimicrobial and bactericidal, with MICs equal to or less than eutherian peptides such as cow BMAP-27 and -28 (87) and human LL-37, including against methicillin-resistant *S. aureus* (88). In particular, MyfaCath7, PspeCath1, PegaCath4, and AnstCath5 had potent, broad-spectrum, and rapid antibacterial activity and are ideal targets for further development (**Table 3**, **Figures 4**–**6**). Marsupial cathelicidins previously found to be antimicrobial also clustered within clade II in the phylogenetic tree. This includes Tasmanian devil SahaCath5 and 6, which are antimicrobial and toxic to Tasmanian devil facial tumor disease (89), and koala PhciCath5, which killed *Chlamydia in vitro* (43). Predicted ancestors of classic marsupial cathelicidins were also antimicrobial (**Supplementary Figure 2**), particularly MAP8, 10, and 14, which were active against all strains tested and killed bacteria within an hour of treatment (**Table 3**, **Figure 4**).

Despite this clear shared antimicrobial function amongst classic marsupial and monotreme cathelicidins, not all peptides displayed activity in our assays as found previously (33, 42, 43). These peptides may be immunomodulatory like many eutherian cathelicidins (8, 9). The immunomodulatory function of marsupial cathelicidins is unknown. However, human LL-37 is expressed by neutrophils, one of the main cells of innate immunity, and is involved in pro- and anti-inflammatory signaling by binding directly to toll-like receptors (TLR) or sequestering lipopolysaccharide and thereby preventing TLR binding (8, 9). Alternatively, these marsupial cathelicidins may be active against strains not tested, such as the many bacterial genera identified within the pouch microbiome. The pouch contains diverse bacterial communities, with numerous phyla identified, including Bacteroidetes, Proteobacteria, Actinobacteria, and Firmicutes (35, 90). While *Staphylococcus*, *Streptococcus*, and *Escherichia* tested in our assays have been identified in the pouch (33, 35), marsupial cathelicidins may have evolved activity against specific pouch bacteria. For example, bacterial families Muribaculaceae and Enterobacteriaceae dominated the pouch microbiome of koalas with healthy and unhealthy young, respectively (90). Cathelicidins within the milk and mammary glands may be active against strains such as Actinobacteria, the predominant phyla in the southern hairy-nosed wombat milk microbiome (35). Future work to screen marsupial cathelicidins against bacteria from these families and others may uncover additional peptides with antimicrobial activity.

Our results suggest a shared evolutionary history and antimicrobial function amongst classic cathelicidins in monotremes, marsupials, and eutherians. These cathelicidins likely evolved from a common ancestor prior to the divergence of monotremes from therian mammals around 180 MYA (30). It is not surprising that the marsupial and monotreme cathelicidins in clade II are antimicrobial, as they share many physiochemical properties with eutherian cathelicidins that are indicative of this function, such as high cationic charge and amphipathicity (7). Indeed, high cationic charge and high percentage of cationic residues were significant predictors of antimicrobial activity amongst marsupial and monotreme cathelicidins in this study, as found for eutherians (7). This suggests marsupial and monotreme cathelicidins likely use electrostatic interaction between positively charged peptides and negatively charged microbial membranes to exert their antimicrobial function (7). However, many marsupial cathelicidins were resistant to the inhibitory effects of divalent cations that compete with the cationic peptides for microbial binding sites (91, 92). As such, marsupial cathelicidins may also utilize alternative mechanisms such as the inhibition of DNA replication (93, 94) or protein translation (95).

### 4.2 Duplication of neutrophil granule protein in marsupials

Other marsupial and monotreme cathelicidins were more closely related to eutherian *NGP* and chicken cathelicidins than eutherian cathelicidins. These cathelicidins sit within clade I in the phylogenetic tree and were encoded in cluster B in the genome of all species studied (**Figure 3**), syntenic to eutherian *NGP* and chicken *cathelicidins* (**Figure 2**). *NGP* is a member of the cystatin superfamily, as are cathelicidins, and has been identified in many eutherians, but is absent in dogs and primates (96, 97). We have identified *NGP* orthologs in 16 marsupial species and platypus, including genes previously characterized as cathelicidins in the koala [*PhciCath6* (43)], opossum [*ModoCath8* (44)], and platypus [*OranCath10* (30)]. Marsupial and monotreme *NGP* contained the same modifications to cathelin domain motif 1 as eutherian *NGP* (**Supplementary Figure 4**) and were encoded in a region of the genome syntenic to *NGP* in mouse and cow (**Figure 2**). As such, we are confident these genes are *NGP* and not cathelicidins. *NGP* is encoded as a single gene copy in all eutherian species studied (25), but this may not be the case in marsupials. Up to five putative *NGP* gene copies were identified within an individual species amongst all 18 marsupials included in this study (**Figure 3**; **Supplementary Table 3**). Putative *NGP* gene duplicates were encoded upstream of the *NGP* ortholog within the genome of all species (**Figure 2**) and clustered within the NGP-like clade in **Figure 3**. This suggests *NGP* has likely evolved through lineage-specific gene duplication in marsupials, but not eutherians or platypus. Initially, these NGP and NGP-like genes were thought to be cathelicidins as they contained the same cathelin domain cysteine motif and other sequence features. However, the phylogeny, combined with genomic organization, indicates these genes are in fact multiple copies of *NGP*.

The multiple *NGP* gene copies in marsupials are likely functional, as they were expressed in immune, reproductive, circulatory, and respiratory tissues, as well as koala milk (47). This includes tammar wallaby *MaeuCath9* that was widely expressed in pouch young tissues from day 1 after birth and throughout development, and hence must have an essential function (39). The function of eutherian *NGP* is not well understood but is likely immunomodulatory and not antimicrobial. Mouse *NGP* regulates pro-inflammatory cytokine expression (28) and has anti-cancer activity (98). Unlike cathelicidins, *NGP* does not undergo post-translational enzymatic cleavage and the C-terminal peptide has a net negative charge (96, 97). Our results concur, as predicted MPs from marsupial and platypus *NGP* orthologs had low or neutral charge at pH 7 (average +1.97) and none displayed antimicrobial activity. This was also the case for MAP1, the predicted ancestral cathelicidin of the NGP clade. However, predicted MPs from putative marsupial *NGP* gene duplicates had a high cationic charge (average +8.6) which indicates antimicrobial potential, and one displayed activity in our assays (*TrvuCath2*). Marsupials and platypus likely encode multiple copies of *NGP*, some of which may have antimicrobial activity, unlike the single *NGP* in eutherians. Future research is required to understand the functional role of multiple *NGP* genes in marsupials, and whether this duplication may be linked to their unique life history as for cathelicidins.

### 4.3 Non-classical cathelicidins in marsupials and monotremes

Alongside classic cathelicidins and NGP, marsupials and monotremes may also encode non-classical cathelicidins as found in birds (23, 27). These cathelicidins cluster with chicken cathelicidins or form a basal clade in the phylogenetic tree (**Figure 3**) and include genes from 15 marsupial species and two monotremes. All putative non-classical cathelicidins were encoded in cluster B in the genome of all marsupials and monotremes, downstream of *NGP* (**Figure 2**). These cathelicidins were expressed in numerous tissues, although many were partial sequences missing the mature peptide (**Supplementary Table 3**). Despite this, 50% of the marsupial and monotreme non-classical cathelicidins tested to date have antimicrobial activity, including the proline/arginine rich *AnstCath2* and *MyfaCath3*, and arginine rich *SmcrCath12* identified in this study (**Table 3**). Additional work is required to determine if non-classical cathelicidins in marsupials display potent and broad-spectrum antimicrobial activity similar to those in chickens (23). The continued annotation of cathelicidins in other marsupials, particularly species from the remaining four extant families not included in this study, will also ensure that our results are not an artifact of missing data within the phylogenetic tree.

While cathelicidins have been characterized across vertebrates (17, 23, 38, 74, 99, 100), the evolutionary history of this gene family is unknown. One theory is that cathelicidins in birds and mammals evolved from an ancestral gene prior to the separation of these lineages (23). Classic cathelicidins likely evolved after mammals diverged from birds, as they have not been identified in birds to date (23, 27, 101). Our study supports this theory, as we show that classic cathelicidins are found in all three lineages of mammals (monotremes, marsupials, and eutherians) and have a conserved genomic organisation and antimicrobial function. In birds, non-classical cathelicidins are thought to represent an extant relative of the ancestral vertebrate cathelicidin, which has been lost in eutherian mammals (23). Our results suggest that marsupials and monotremes may also encode non-classical cathelicidins, unlike eutherians, although additional work is required for confirmation. The marsupial and monotreme cathelicidin gene repertoires reflect aspects of both birds (non-classical *cathelicidins*) and eutherians (classical *cathelicidins* and *NGP*) and may represent an evolutionary stepping stone between these two lineages.

Marsupials have a large and diverse toolkit of cathelicidin NGP genes resulting from lineage-specific gene duplication. Classic marsupial cathelicidins and their predicted ancestors have potent and rapid antimicrobial activity that could be utilized for future therapeutic development. Marsupials also encode genes similar to non-classical cathelicidins and multiple gene copies of *NGP*, unlike eutherians. Future work should focus on exploring the immunomodulatory function of marsupial cathelicidins and cathelicidin-like peptides and confirming the presence and biological significance of non-classical cathelicidins and NGP gene duplicates in marsupials. Our results not only shed light on cathelicidin evolution amongst mammals, but also highlight the therapeutic potential of marsupial cathelicidins and ASR as a tool to design potent antimicrobials.

## Data Availability

Blood transcriptomes for mahogany glider, southern hairy nosed wombat and rufous hare wallaby generated in this study are available through Australasian Genomes Amazon Web Services open datasets program (https://registry.opendata.aws/australasian-genomics/). The raw RNAseq reads have been accessioned with NCBI under BioProject PRJNA1177593. All other genomes and transcriptomes used in this project are publicly available through NCBI, DNA Zoo or Australasian Genomes Amazon Web Services Open Datasets Program. See **Supplementary Table 1** for accession numbers.
