# Supplementary material for: Marsupial cathelicidins: characterization, antimicrobial activity and evolution in this unique mammalian lineage
Source: Front Immunol. 2025 Apr 4;16:1524092. doi: 10.3389/fimmu.2025.1524092 (PMC12006171; doi:10.3389/fimmu.2025.1524092)
Supplement: Supplementary file 1 [file DataSheet1.docx]

***Supplementary Material***

[1 Supplementary information 2](#_Toc194478145)

[Blood transcriptome assembly & annotation 2](#_Toc194478146)

[2 Supplementary tables 3](#_Toc194478147)

[Supplementary table 1. 3](#_Toc194478148)

[Supplementary table 2. 5](#_Toc194478149)

[Supplementary table 3. 5](#_Toc194478150)

[Supplementary table 4. 6](#_Toc194478151)

[Supplementary table 5 6](#_Toc194478152)

[Supplementary table 6. 7](#_Toc194478153)

[Supplementary table 7 8](#_Toc194478154)

[Supplementary table 8. 9](#_Toc194478155)

[Supplementary table 9 11](#_Toc194478156)

[Supplementary table 10. 11](#_Toc194478157)

[Supplementary table 11 12](#_Toc194478158)

[3 Supplementary figures 13](#_Toc194478159)

[Supplementary figure 1 13](#_Toc194478160)

[Supplementary figure 2. 14](#_Toc194478161)

[Supplementary figure 3. 15](#_Toc194478162)

[Supplementary figure 4. 16](#_Toc194478163)

[4 References 17](#_Toc194478164)

# 1 Supplementary information

## Blood transcriptome assembly & annotation

Here we report the first marsupial blood transcriptomes, and the first omics data available for the mahogany glider. *De novo* blood transcriptomes for the mala, southern hairy-nosed wombat and mahogany glider contained over 200k transcripts within each assembly, with a transcript N50 of 1.2 to 1.3kbp and an average transcript length of 674 to 681bp (supplementary table 2). All three assemblies displayed a high mapping rate of up to 98.96%, indicating they accurately represented trimmed input reads. The percentage of complete vertebrate BUSCO genes within each of the assemblies ranged from 66-75%, however values were lower when searching the mammalian lineage. While this indicates the assemblies are missing single copy gene orthologs, the BUSCO and *de novo* assembly statistics for these marsupial blood transcriptomes are similar to other *de novo* wildlife blood transcriptomes from the beluga whale (1), bottlenose dolphin (1) and greater mouse-eared bat (2). Up to 13,564 unique Swiss-Prot genes were annotated within each of the three assemblies (supplementary table 2).

# 2 Supplementary tables

Supplementary table 1. Genomic and transcriptomic datasets used in this study, including NCBI accession numbers. *Eastern barred bandicoot genome data was downloaded from the Oz Mammals Genomics Initiative Bioplatforms Australia data portal. ^+^Brushtail possum genome accessioned with NCBI has not been published. ^1^datasets available through Australasian Genomes on Amazon Web Services open datasets program (<https://registry.opendata.aws/australasian-genomics>). ^2^The common wombat genome annotation is available through DNA Zoo (<https://www.dnazoo.org/assemblies/vombatus_ursinus>)

| **Species** | **Data type** | **Genome**  **assembly** | **Genome annotation** | **Transcriptome** |
| --- | --- | --- | --- | --- |
| Brown antechinus (*Antechinus stuartii*) | Genome & 10 transcriptomes | GCA_016696395.1 (3) | antechinusM_pseudohap2.1.gff3 (4) | PRJNA664282 (3) |
| Fat-tailed dunnart (*Sminthopsis crassicaudata*) | Genome & 1 transcriptome | Available through Figshare (5) | Available through Figshare (5) | PRJNA399240 (6) |
| Numbat (*Myrmecobius fasciatus*) | Genome & 3 transcriptomes | GCA_023553655.1 (7) | mMyrfas1.pri.20211206.gff3^1^ (8) | PRJNA786364 (7) |
| Greater bilby (*Macrotis lagotis*) | Genome & 12 transcriptomes | GCA_037893015.1 (9) | bilby.v1.5.hirise.ensrep200kb.gff  (9) | PRJNA1049866 (9) |
| Brushtail possum (*Trichosurus vulpecula*) | Genome & 2 transcriptomes | GCF_011100635.1^+^ | NCBI RefSeq annotation | PRJNA525264 (10) |
| Eastern grey kangaroo (*Macropus giganteus*) | Genome | DNA Zoo (11, 12) | Not available | N/A |
| Red Kangaroo (*Macropus rufus*) | Genome | DNA Zoo (11, 12) | Not available | N/A |
| Rufous hare wallaby (*Lagorchestes hirsutus*) | Genome & blood transcriptome | DNA Zoo GCA_028533205.1 (11, 12) | Not available | This study |
| Eastern barred bandicoot (*Perameles gunnii*) | Genome | Not published* | Not available | N/A |
| Mahogany glider (*Petaurus gracilis*) | Blood transcriptome | N/A | N/A | This study |
| Western ringtail possum (*Pseudocheirus peregrinus*) | Genome | DNA Zoo (11, 12) | Not available | N/A |
| Southern hairy nosed wombat (*Lasiorhinus latifrons*) | Blood transcriptome | N/A | N/A | This study |
| Common wombat (*Vombatus ursinus*) | Genome | DNA Zoo GCA_028626985.1 (11, 12) | vu-2k.fasta_v2.functional.gff3^2^(12) | N/A |
| Woylie (*Bettongia penicillata ogilbyi*) | Genome & 4 transcriptomes | GCA_023548195.1 (13) | mBetpen1.pri.20210916.gff3^1^ (14) | PRJNA763700 |
| Tasmanian devil (*Sarcophilus harrisii*) | Genome | GCA_902635505.1 | NCBI RefSeq annotation | N/A |
| Tammar wallaby (*Notamacropus eugenii*) | Genome | GCA_028372415.1 | Not available | N/A |
| Koala (*Phascolarctos cinereus*) | Genome | GCA_003287225.2 | Not available | PRJNA230900 & PRJNA327021 (15, 16) |
| Gray short-tailed opossum (*Monodelphis domestica*) | Genome | GCF_027887165.1 | NCBI RefSeq annotation | N/A |

Supplementary table 2. Accession numbers for eutherian, avian and fish cathelicidin and neutrophil granule protein (NGP) sequences used in this study.

| **Species** | **Gene** | **Accession** |
| --- | --- | --- |
| Human (*Homo sapiens*) | CAMP | P49913 (UniProt) |
| Mouse (*Mus musculus*) | CRAMP | P51437 (UniProt) |
|  | NGP | O08692 (UniProt) |
| Rat (*Rattus rattus*) | NGP | D3ZY96 (UniProt) |
| Rabbit (*Oryctolagus cuniculus*) | P15A | P26202 (UniProt) |
|  | P15B | P26203 (UniProt) |
| Cow (*Bos taurus*) | BAMP-27 (Cath6) | P54228 (UniProt) |
|  | BMAP-28 (Cath5) | P54229 (UniProt) |
|  | BMAP-34 (Cath7) | P56425 (UniProt) |
|  | Bac7 (Cath3) | P19661 (UniProt) |
|  | Bac5 (Cath2) | P19660 (UniProt) |
|  | Bac1 | P22226 (UniProt) |
|  | Indolicidin (Cath4) | P33046 (UniProt) |
|  | NGP | ENSBTAT00000044676.4 (Ensembl) |
| Pig (*Sus scrofa*) | PMAP-37 | P49932 (UniProt) |
|  | PMAP-23 | P49930 (UniProt) |
|  | PMAP-36 | P49931 (UniProt) |
|  | Protegrin-1 | P32194 (UniProt) |
|  | Protegrin-2 | P32195 (UniProt) |
|  | Protegrin-3 | P32196 (UniProt) |
|  | Protegrin-5 | P49934 (UniProt) |
|  | PR-39 | P80054 (UniProt) |
| Sheep (*Ovis aries*) | Bac5 (Cath2) | P79362 (UniProt) |
|  | Bac1A (Cath1A) | P54230 (UniProt) |
|  | Bac1B | NM_001009772.1 (GenBank) |
|  | SMAP-29 | P49929 (UniProt) |
|  | MAP-34 | P79360 (UniProt) |
|  | Bac7 (Cath3) | P50415 (UniProt) |
| Chicken (*Gallus gallus*) | Cath1 | Q6QLQ5 (UniProt) |
|  | Cath2 | Q2IAL7 (UniProt) |
|  | Cath3 | Q2IAL6 (UniProt) |
| Atlantic salmon (*Salmo salar*) | Cath1 | AY728057 (GenBank) |
|  | Cath2 | AY360357 (GenBank) |
| Rainbow trout (*Oncorhynchus mykiss*) | Cath1 | AY382478.1 (GenBank) |
|  | Cath2 | AY360356 (GenBank) |

Supplementary table 3. Genome and/or transcriptome annotation of cathelicidins in the 18 species included in this study. Predicted mature peptide sequences and physiochemical properties are also included. See excel spreadsheet *Supplementary_table_3.xls*

Supplementary table 4. Strain information for all bacterial and fungal isolates used in this study

| **Isolate** | **Source** |
| --- | --- |
| *Staphylococcus aureus* | ATCC 29213 |
| *Staphylococcus aureus* | clinical isolate - animal |
| *Staphylococcus aureus* | clinical isolate – animal, methicillin-resistant |
| *Staphylococcus aureus* | clinical isolate – animal, penicillin-resistant |
| *Escherichia coli* | ATCC 25922 |
| *Escherichia coli* | clinical isolate - animal |
| *Pseudomonas aeruginosa* | ATCC 27853 |
| *Pseudomonas aeruginosa* | clinical isolate - animal |
| *Streptococcus agalactiae* | ATCC 12386 |
| *Streptococcus agalactiae* | Clinical isolate - animal |
| *Streptococcus pneumoniae* | ATCC 49619 |
| *Candida krusei* | ATCC 6258 |
| *Candida parapsilosis* | ATCC 22019 |
| *Candida albicans* | Clinical isolate - human |

Supplementary table 5. Assembly statistics for rufous hare wallaby (mala), southern hairy-nosed wombat and mahogany glider blood transcriptomes generated in this study.

|  | **Rufous hare wallaby** | **Southern hairy nosed wombat** | **Mahogany glider** |
| --- | --- | --- | --- |
| No. transcripts | 252,824 | 378,033 | 214,833 |
| GC (%) | 44.68 | 44.39 | 45.64 |
| Transcript N50 | 1,383 | 1,280 | 1,297 |
| Avg. transcript length (bp) | 674.9 | 681.50 | 672.25 |
| Complete BUSCOs v5:  Mammalian | 48.2% | 56.6% | 47.8% |
| Vertebrate | 66.3% | 75.8% | 64.4% |
| Read representation (%) | 98.30 | 97.50 | 98.96 |
| No. unique Swiss-Prot genes annotated by Trinotate | 12,770 | 13,564 | 12,626 |

Supplementary table 6. Comparison of cathelicidin genes annotated in different versions of the opossum, Tasmanian devil, koala, and tammar wallaby genomes. Cathelicidins have been annotated in the latest genome assembly of the platypus and echidna, however genomic organisation was not investigated in this publication. NCBI accession numbers are provided for each genome assembly, as well as the assembly name and year it was accessioned with NCBI. Investigation of cathelicidin gene cluster genomic organisation and synteny within each assembly is outlined.

| **Species** | **Genome assembly version** | **Number of cathelicidin genes** | **Genomic organisation & synteny?** |
| --- | --- | --- | --- |
| Opossum  (*Monodelphis domestica*) | MonDom4 (2007)  GCA_000002295.1 | 19 (17, 18) | Y (organisation) |
|  | MonDom1 (2023) GCA_027887165.1 | 15 (this study) | Y (this study) |
| Tasmanian devil  (*Sarcophilus harrisii*) | Devil_ref v7.0 (2011) GCA_000189315.1 | 7 (19, 20) | Y (organisation) |
|  | mSarHar1.11 (2019)  GCA_902635505.1 | 7 (this study) | Y (this study) |
| Koala  (*Phascolarctos cinereus*) | phaCin_unsw_v4.1 (2017)  GCA_002099425.1 | 10 (21) | Y (organisation) |
|  | PhaCin_HiC (2022) GCA_003287225.2 | 10 (this study) | Y (this study) |
| Tammar wallaby  (*Notamacropus eugenii*) | Meug_1.1 (2011) GCA_000004035.1 | 8 | N |
|  | MacEug1 (2023) GCA_028372415.1 | 18 (this study) | Y (this study) |
| Platypus  (*Ornithorhynchus anatinus*) | mOrnAna1 (2021)  GCF_004115215.2 | 10 (22) | N |
| Echidna  (*Tachyglossus aculeatus*) | mTacAcu1 (2021)  GCF_015852505.1 | 6 (22) | N |

Supplementary table 7**.** Comparison of cathelicidin gene cluster size size (kb) and gene number in the genomes of the ten marsupials, two monotremes and four eutherian species included in the gene cluster comparison. Species with draft-quality genomes (numbat, eastern grey kangaroo, red kangaroo, western ringtail possum, common wombat and rufous hare wallaby) were not included in this comparison due to the high number of orphan cathelicidin genes identified outside the main scaffold. The mahogany glider was also excluded from this analysis as a genome is not available for this species.

| **Species** | **Cluster A** | | **Cluster B** | | **Cluster C** | |
| --- | --- | --- | --- | --- | --- | --- |
|  | Gene number | Size (kb) | Gene number | Size (kb) | Gene number | Size (kb) |
| Human | 1 | 2 | N/A | N/A | N/A | N/A |
| Mouse | 1 | 2 | N/A | N/A | N/A | N/A |
| Cow | 7 | 98 | N/A | N/A | N/A | N/A |
| Sheep | 6 | 65 | N/A | N/A | N/A | N/A |
| Gray short-tailed opossum | 4 | 128 | 8 | 262 | N/A | N/A |
| Tasmanian devil | 4 | 76 | 3 | 90 | N/A | N/A |
| Fat-tailed dunnart | 7 | 126 | 8 | 184 | N/A | N/A |
| Brown antechinus | 5 | 88 | 4 | 88 | N/A | N/A |
| Greater bilby | 4 | 52 | 5 | 181 | N/A | N/A |
| Eastern barred bandicoot | 6 | 91 | 4 | 226 | N/A | N/A |
| Brushtail possum | 7 | 158 | 7 | 162 | N/A | N/A |
| Koala | 6 | 143 | 3 | 57 | N/A | N/A |
| Woylie | 4 | 179 | 4 | 251 | N/A | N/A |
| Tammar wallaby | 10 | 293 | 8 | 192 | N/A | N/A |
| Platypus | 3 | 29 | 2 | 14 | 5 | 33 |
| Short beaked echidna | 2 | 89 | 1 | 15 | 3 | 32 |

Supplementary table 8. MIC µM equivalent for MIC values in table 3 in the main manuscript. Peptides with the most potent and/or broad-spectrum activity are highlighted in grey. For explanation of superscript see table 3 in the main text.

| **Species** | **Mature peptide** | ***S. aureus* ATCC** | ***S. aureus*** | ***E. coli* ATCC** | ***E. coli*** | ***P. aeruginosa* ATCC** | ***P. aeruginosa*** | ***S. agalactiae* ATCC*** | ***S. agalactiae**** | ***S. pneumoniae* ATCC*** | **MRSA^+^** | **PRSA^+^** |
| --- | --- | --- | --- | --- | --- | --- | --- | --- | --- | --- | --- | --- |
| Antechinus | AnstCath2 | >18.2 | >18.2 | 4.5 (4.5) | 9.1 (18.2) | >18.2 | >18.2 | N/A | N/A | N/A | N/A | N/A |
|  | AnstCath4 | 13.5 (13.5)^13.5^ | 13.5 (13.5)^13.5^ | 27.1 (27.1)^27.1^ | 13.5 (27.1)^27.1^ | >27.1 | >27.1 | >27.1 | >27.1 | >27.1 | 13.5 (27.1) | 6.7 (6.7) |
|  | AnstCath5 | 4.4 (8.9)^4.4^ | 8 (4.4)^2.2^ | 4.4 (8.9)^4.4^ | 8.9 (17.9)^8.9^ | >17.9 | 8.9 (17.9)^17.9^ | >17.9 | 8.9 (8.9) | >17.9 | 4.4 (8.9) | 4.4 (4.4) |
| Fat-tailed dunnart | SmcrCath3 | 6.5 (6.5)^6.5^ | 6.5 (6.5)^6.5^ | 6.5 (6.5)^13.1^ | 3.2 (6.5)^6.5^ | >26.3 | 26.3 (>26.3)^>27.3^ | >26.3 | >26.3 | >26.3 | 6.5 (6.5) | 3.2 (6.5) |
|  | SmcrCath6 | >22.6 | >22.6 | 11.3 (22.6)^22.6^ | >22.6 | >22.6 | >22.6 | >22.6 | >22.6 | >22.6 | N/A | N/A |
|  | SmcrCath12 | >15.6 | >15.6 | 1.9 (1.9) | 3.9 (3.9) | >15.6 | >15.6 | N/A | N/A | N/A | N/A | N/A |
| Numbat | MyfaCath3 | >13.6 | >13.6 | 1.7 (1.7) | 3.4 (3.4) | >13.6 | >13.6 | N/A | N/A | N/A | N/A | N/A |
|  | MyfaCath5 | 8.8 (17.7)^4.4^ | 4.4 (8.8)^4.4^ | 8.8 (17.7)^8.8^ | 8.8 (8.8)^8.8^ | >17.7 | >17.7 | >17.7 | >17.7 | >17.7 | 8.8 (8.8) | 2.2 (2.2) |
|  | MyfaCath7 | 3.4 (3.4)^3.4^ | 3.4 (3.4)^3.4^ | 1.7 (1.7)^3.4^ | 1.7 (1.7)^1.7^ | >27.4 | 13.7 (>27.4)^27.4^ | >27.4 | 13.7 (13.7) | >27.4 | 3.4 (3.4) | 1.7 (1.7) |
| Eastern grey kangaroo | MagiCath3 | >14.3 | >14.3 | 14.3 (>14.3) | >14.3 | >14.3 | >14.3 | N/A | N/A | N/A | N/A | N/A |
|  | MagiCath6 | 17.6 (17.6) | 17.6 (17.6) | 8.8 (8.8) | 17.6 (17.6) | >17.6 | >17.6 | N/A | N/A | N/A | N/A | N/A |
| Rufous hare wallaby | LalaCath3 | 2 (4.1)^2^ | 2 (4.1)^2^ | 8.3 (8.3)^8.3^ | 16.6 (16.6)^>16.6^ | >16.6 | >16.6 | >16.6 | 8.3 (8.3) | >16.6 | 2 (4.1) | 1 (2) |
| Red kangaroo | MaruCath4 | >13.2 | >13.2 | 1.6 (1.6)^1.6^ | 6.6 (6.6)^6.6^ | >13.2 | >13.2 | >13.2 | >13.2 | >13.2 | N/A | N/A |
|  | MaruCath9 | 8.6 (8.6) | 8.6 (8.6) | >17.3 | >17.3 | >17.3 | >17.3 | N/A | N/A | N/A | N/A | N/A |
| Eastern barred bandicoot | PeguCath3 | >14.1 | >14.1 | 14.1 (14.4) | >14.1 | >14.1 | >14.1 | N/A | N/A | N/A | N/A | N/A |

| Mahogany glider | PegaCath4 | 9 (>18)^18^ | 18 (>18)^18^ | 1.1 (2.2)^1.1^ | 1.1 (1.1)^0.5^ | 2.2 (4.5)^2.2^ | 2.2 (4.5)^2.2^ | >18 | >18 | >18 | 18 (>18) | 4.5 (>18) |
| --- | --- | --- | --- | --- | --- | --- | --- | --- | --- | --- | --- | --- |
| Brushtail possum | TrvuCath2 | >17.5 | >17.5 | 17.5 (17.5)^17.5^ | 17.5 (17.5)^17.5^ | >17.5 | >17.5 | >17.5 | >17.5 | >17.5 | N/A | N/A |
| Woylie | BepeCath4 | 7.9 (15.9) | 7.9 (7.9) | 3.9 (3.9) | 3.9 (7.9) | >15.9 | >15.9 | N/A | N/A | N/A | N/A | N/A |
|  | BepeCath7 | >13.2 | >13.2 | 6.6 (13.2)^6.6^ | 6.6 (13.2)^6.6^ | >13.2 | >13.2 | >13.2 | >13.2 | >13.2 | N/A | N/A |
| Western ringtail possum | PspeCath1 | >17.5 | >17.5 | 1 (2.1)^1^ | 1 (1)^0.5^ | 2.1 (2.1)^2.1^ | 2.1 (4.3)^2.1^ | >17.5 | >17.5 | >17.5 | N/A | N/A |
| Bilby | MacroCath7 | >16.3 | >16.3 | 8.1 (16.3)^8.1^ | 16.3 (16.3)^16.3^ | >16.3 | >16.3 | >16.3 | >16.3 | >16.3 | N/A | N/A |
|  | MacroCath8 | >13.6 | >13.6 | >13.6 | 13.6 (>13.6)^13.6^ | >13.6 | >13.6 | >13.6 | >13.6 | >13.6 | N/A | N/A |
| Southern hairy nosed wombat | LahiCath4 | >14.5 | >14.5 | 7.2 (14.5)^14.5^ | 3.6 (14.5)^14.5^ | >14.5 | >14.5 | >14.5 | >14.5 | >14.5 | N/A | N/A |
|  | LahiCath5 | >15.7 | >15.7 | 3.9 (3.9) | 3.9 (7.8) | >15.7 | >15.7 | N/A | N/A | N/A | N/A | N/A |
| Predicted ancestral cathelicidin | MAP6 | >21.8 | >21.8 | 10.9 (10.9)^10.9^ | 10.9 (10.9)^10.9^ | >21.8 | >21.8 | >21.8 | >21.8 | >21.8 | N/A | N/A |
|  | MAP7 | >15.1 | >15.1 | 1.8 (3.7)^1.8^ | 3.7 (3.7)^3.7^ | 7.5 (7.5)^7.5^ | 7.5 (64)^7.5^ | 15.1 (15.1) | 15.1 (15.1) | 7.5 (7.5) | N/A | N/A |
|  | MAP8 | 7.4 (7.4)^7.4^ | 7.4 (7.4)^7.4^ | 3.7 (7.4)^3.7^ | 3.7 (3.7)^1.8^ | 3.7 (3.7)^3.7^ | 3.7 (7.4)^3.7^ | 14.8 (14.8) | 7.4 (7.4) | 7.4 (7.4) | 14.8 (>14.8) | 3.7 (7.4) |
|  | MAP10 | 4.1 (8.2)^4.1^ | 4.1 (4.1)^4.1^ | 2 (4.1)^8^ | 2 (2)^2^ | 4.1 (4.1)^4.1^ | 4.1 (8.2)^4.1^ | 16.4 (16.4) | 16.4 (16.4) | 16.4 (>16.4) | 4.1 (8.2) | 2 (4.1) |
|  | MAP11 | 16 (9.3)^9.3^ | 9.3 (9.3)^4.6^ | 9.3 (18.6)^9.3^ | 9.3 (9.3)^18.6^ | >18.6 | >18.6 | >18.6 | >18.6 | >18.6 | 9.3 (9.3) | 4.6 (4.6) |
|  | MAP13 | >15.4 | >15.4 | 7.7 (7.7)^7.7^ | 7.7 (7.7)^7.7^ | 7.7 (15.4)^15.4^ | 15.4 (15.4)^15.4^ | >15.4 | >15.4 | >15.4 | N/A | N/A |
|  | MAP14 | 7.9 (7.9)^7.9^ | 7.9 (7.9)^7.9^ | 1.9 (1.9)^1.9^ | 1.9 (1.9)^0.9^ | 3.9 (3.9)^1.9^ | 3.9 (3.9)^3.9^ | 15.8 (15.8) | 7.9 (7.9) | 7.9 (7.9) | 15.8 (15.8) | 3.9 (3.9) |
| Controls | Ampicillin | 2.8^5.7^ | 0.7^0.7^ | 11.4^22.8^ | >183.1 | N/A | N/A | 0.7 | 0.7 | >183.1 | N/A | N/A |
|  | Tetracycline | N/A | N/A | N/A | N/A | 72^72^ | 72^72^ | N/A | N/A | N/A | N/A | N/A |

Supplementary table 9**.** Number of hours for cathelicidins to cause a significant reduction in log CFU/mL of *S. aureus* ATCC (*p* < 0.05) at three concentrations: half the MIC (0.5x MIC), the MIC, and two-fold higher than the MIC (2x MIC). For MIC values see table 3. NS indicates the peptide did not cause a significant reduction in bacterial growth at any timepoint tested.

|  | **0.5X MIC** | **MIC** | **2X MIC** |
| --- | --- | --- | --- |
| SmcrCath3 | 4 | 2 | 2 |
| AnstCath5 | 2 | 2 | 2 |
| LalaCath3 | 4 | 4 | 2 |
| Myfacath7 | 2 | 2 | 2 |
| MAP10 | NS | 4 | 4 |
| MAP11 | 4 | 4 | 2 |

Supplementary table 10. Number of hours for cathelicidins to cause a significant reduction in log CFU/mL of *E. coli* ATCC (*p* < 0.05) at three concentrations: half the MIC (0.5x MIC), the MIC, and two-fold higher than the MIC (2x MIC). For MIC values see table 3. NS indicates the peptide did not cause a significant reduction in bacterial growth at any timepoint tested.

|  | **0.5x MIC** | **MIC** | **2X MIC** |
| --- | --- | --- | --- |
| MyfaCath7 | 4 | 4 | 4 |
| PspeCath1 | 2 | 2 | 1 |
| PegaCath4 | 6 | 6 | 6 |
| MaruCath4 | 4 | 4 | 4 |
| AnstCath5 | NS | 4 | 2 |
| SmrcCath3 | 4 | 4 | 2 |
| MAP7 | NS | 4 | 4 |
| MAP8 | 1 | 1 | 1 |
| MAP10 | 4 | 4 | 2 |
| MAP14 | 1 | 1 | 1 |

| **Species** | **Mature peptide** | ***C. parapsilosis* ATCC** | ***C. krusei***  **ATCC** | ***C. albicans*** |
| --- | --- | --- | --- | --- |
| Antechinus | AnstCath4 | 17.9 (>17.9) | 8.9 (17.9) | 17.9 (17.9) |
|  | AnstCath5 | 13.5 (>27.1) | 13.5 (13.5) | 27.1 (>27.1) |
| Fat-tailed dunnart | SmcrCath3 | 13.5 (13.5) | 6.5 (13.5) | 13.5 (26.3) |
| Numbat | MyfaCath5 | 17.7 (17.7) | 4.4 (4.4) | >17.7 |
|  | MyfaCath7 | 13.7 (13.7) | 6.8 (6.8) | 27.4 (27.4) |
| Western ringtail possum | PspeCath1 | 8.75 (>17.5) | >17.5 | >17.5 |
| Predicted ancestral peptide | MAP8 | 7.4 (14.4) | 14.4 (>14.4) | >14.4 |
|  | MAP9 | 16.5 (>16.5) | 8.2 (16.5) | >16.5 |
|  | MAP10 | 8.2 (>16.4) | >16.4 | >16.4 |
|  | MAP13 | 7.7 (>15.4) | >15.4 | >15.4 |
|  | MAP14 | 3.9 (7.9) | 15.8 (>15.8) | >15.8 |
| Control | Amphotericin B | 0.5 | 1 | 0.5 |

Supplementary table 11. Minimum inhibitory concentration (MIC) in µM of extant and predicted ancestral marsupial cathelicidins against three fungi, compared to the control amphotericin B. MP that were active against all three strains are highlighted in grey.

# **3 Supplementary figures**


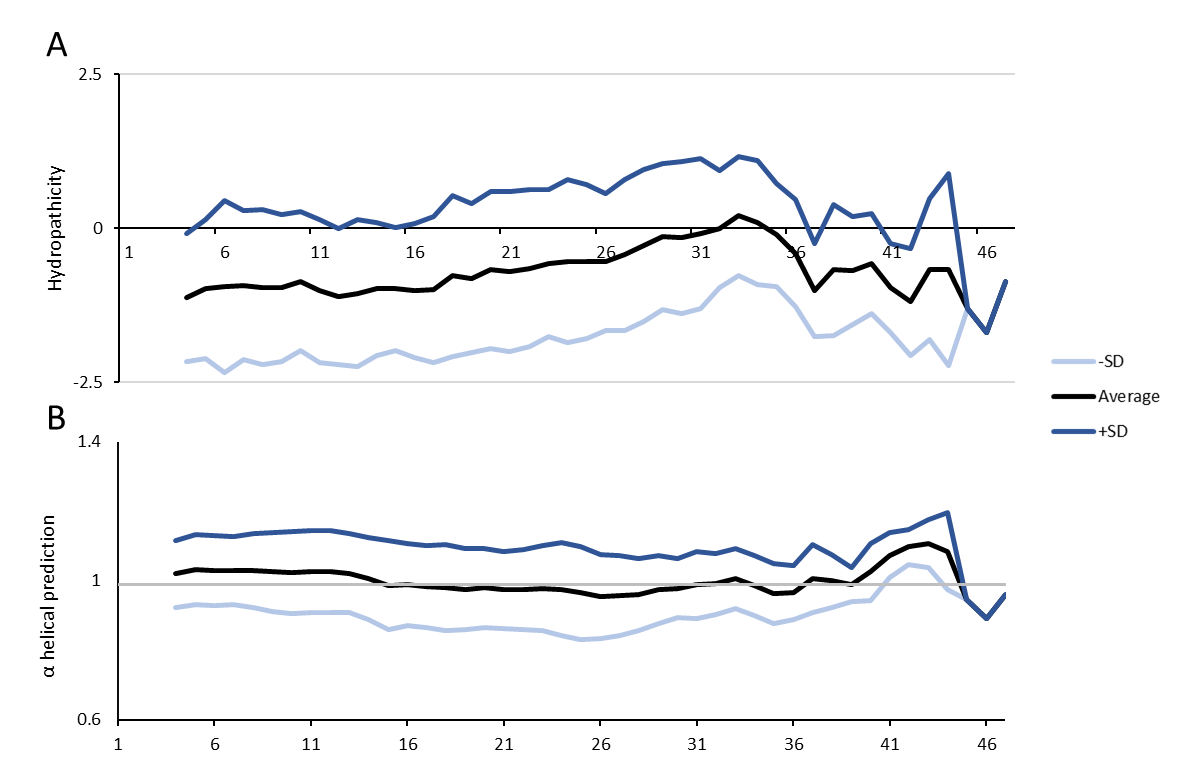
Supplementary figure 1. Average Kyte and Doolittle hydropathicity plot (A) and Deleage and Roux alpha helicity plot (B) for 83 predicted marsupial cathelicidin mature peptides. The standard deviation (SD) is also shown. Positive values in A indicate the presence of hydrophobic regions. Values above the 0.99 cutoff in B (grey line) indicate the presence of alpha helices.


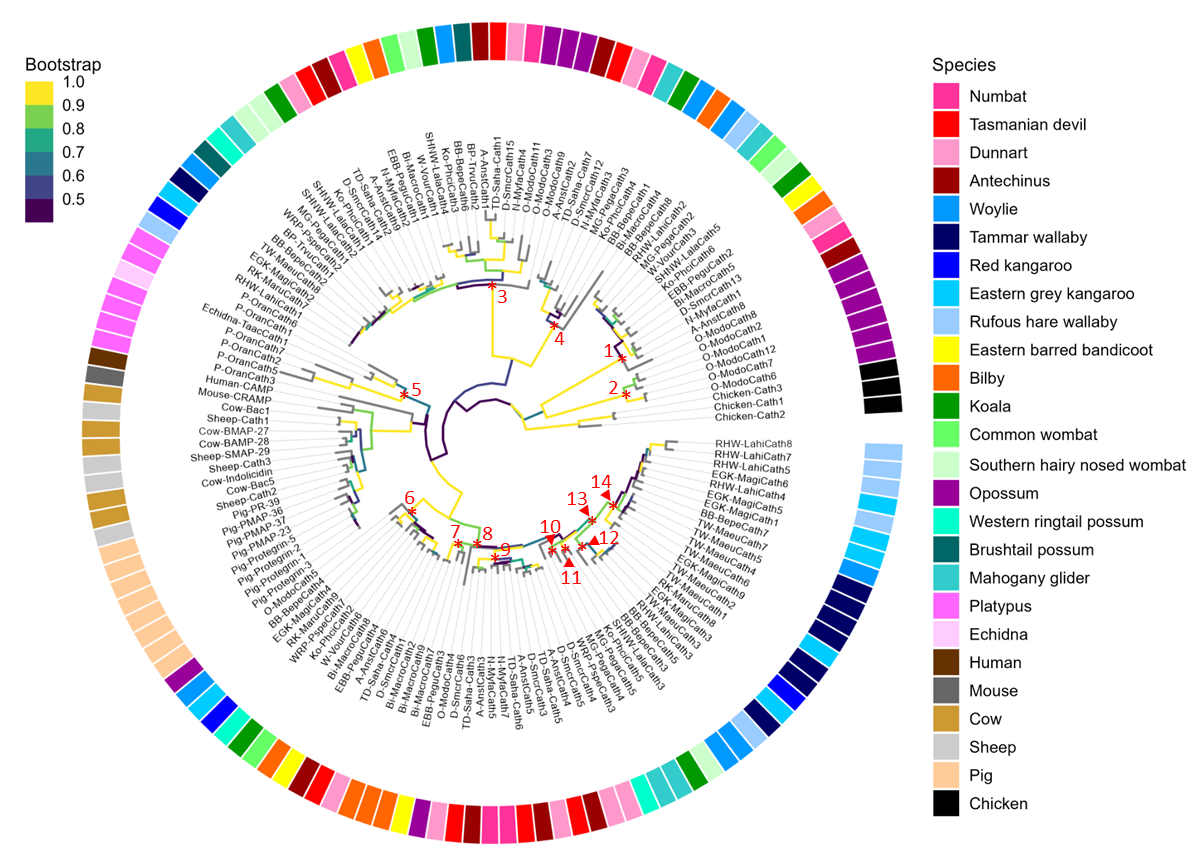
Supplementary figure 2. Maximum-likelihood phylogenetic tree of full-length cathelicidin amino acid sequences from 18 marsupials, two monotremes, five eutherians and chicken. This tree was used for ancestral sequence reconstruction with ANCESCON and GASP. Nodes corresponding to selected ancestral sequences are indicated by * and numbered, which correspond to the marsupial ancestral peptide (MAP1 to MAP14) sequences in supplementary table 1.


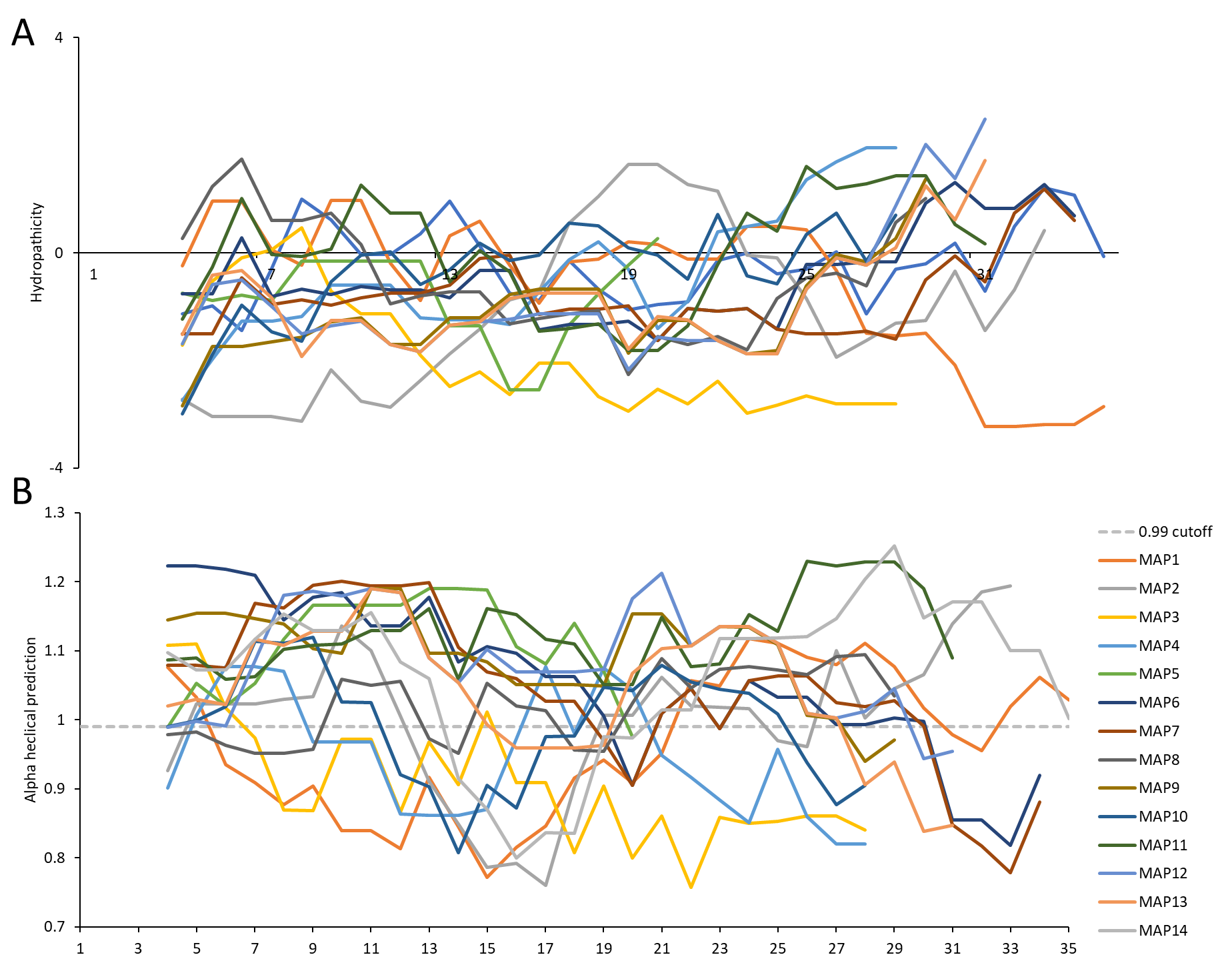
Supplementary figure 3. Kyte and Doolittle hydropathicity plot (A) and Deleage and Roux alpha helicity plot (B) for 14 predicted ancestral marsupial mature peptides. Positive values in A indicate the presence of hydrophobic regions. Values above the 0.99 cutoff in B (grey line) indicate the presence of alpha helices.


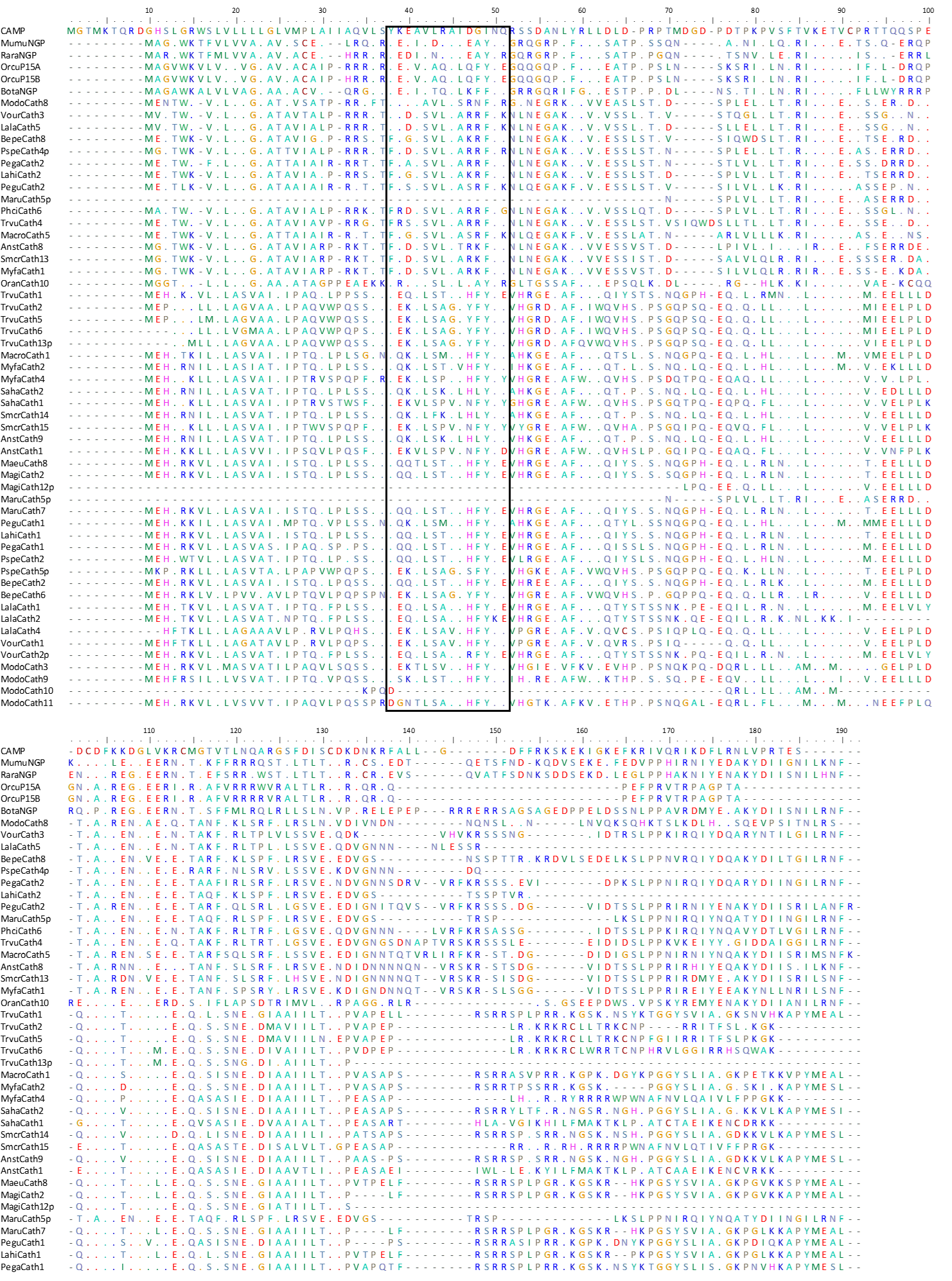
Supplementary figure 4. Protein multiple sequence alignment of the first 100 residues of human cathelicidin (*CAMP)*, eutherian neutrophil granule protein (*NGP*) and putative marsupial and monotreme orthologs. Dots represent residues that are identical to *CAMP*. Cathelicidin motif 1 is boxed. Genes are named according to the first 2 letters of the genus and species.

# 4 References

1. Morey JS, Huntington KAB, Campbell M, Clauss TM, Goertz CE, Hobbs RC, et al. *De novo* transcriptome assembly and RNA-seq expression analysis in blood from beluga whales of Bristol Bay, AK. Marine Genomics. 2017;35:77-92.

2. Huang Z, Gallot A, Lao NT, Puechmaille SJ, Foley NM, Jebb D, et al. A nonlethal sampling method to obtain, generate and assemble whole blood transcriptomes from small, wild mammals. Molecular Ecology. 2016;16:150-62.

3. Brandies PA, Tang S, Johnson RSP, Hogg CJ, Belov K. The first *Antechinus* reference genome provides a resource for investigating the genetic basis of semelparity and age-related neuropathologies. Gigabyte. 2020;2020:0.

4. Brandies PA, Tang S, Johnson RSP, Hogg CJ, Belov K. Supporting data for "The first Antechinus reference genome provides a resource for investgating the genetic basis of semelparity and age-related neuropathologies". GigaScience Database. 2020.

5. Ibeh N, Feigin CY, Frankenberg SR, McCarthy DJ, Pask AJ, Gallego Romero I. De novo transcriptome assembly and genome annotation of the fat-tailed dunnart (Sminthopsis crassicaudata). GigaByte. 2024;2024:gigabyte118.

6. Whittington CM, O’Meally D, Laird MK, Belov K, Thompson MB, McAllan BM. Transcriptomic changes in the pre-implantation uterus highlight histotrophic nutrition of the developing marsupial embryo. Scientific Reports. 2018;8(1):2412.

7. Peel E, Silver L, Brandies PA, Hayakawa T, Belov K, Hogg CJ. Genome assembly of the numbat (*Myrmecobius fasciatus*), the only termitivorous marsupial. Gigabyte. 2022.

8. Peel E, Silver L, Brandies PA, Hayakawa T, Belov K, Hogg CJ. Supporting data for "Genome assembly of the numbat (*Myrmecobius fasciatus*), the only termitivorous marsupial. GigaDB. 2022.

9. Hogg CJ, Edwards RJ, Farquharson KA, Silver LW, Brandies P, Peel E, et al. Extant and extinct bilby genomes combined with Indigenous knowledge improve conservation of a unique Australian marsupial. Nature Ecology & Evolution. 2024;8(7):1311-26.

10. White DJ, Trought K, Hopkins B. The mixed liver and heart transcriptome dataset of the New Zealand brushtail possum, Trichosurus vulpecula. Data in Brief. 2019;27:104577.

11. Dudchenko O, Batra SS, Omer AD, Nyquist SK, Hoeger M, Durand NC, et al. De novo assembly of the *Aedes aegypti* genome using Hi-C yields chromosome-length scaffolds. Science advances. 2017;356:92-5.

12. Dudchenko O, Shamim MS, Batra SS, Durand NC, Musial NT, Mostofa R, et al. The Juicebox Assembly Tools module facilitates *de novo* assembly of mammalian genomes with chromosome-length scaffolds for under $1000. bioRxiv. 2018:254797.

13. Peel E, Silver L, Brandies P, Hogg CJ, Belov K. A reference genome for the critically endangered woylie, *Bettongia penicillata ogilbyi*. Gigabyte. 2021;1.

14. Peel E, Silver L, Brandies PA, Hogg CJ, Belov K. Supporting data for "A reference genome for the critically endanfered woylie, *Bettongia penicillata ogilbyi*". GigaDB. 2021.

15. Hobbs M, Pavasovic A, King AG, Prentis PJ, Eldridge MDB, Chen Z, et al. A transcriptome resource for the koala (*Phascolarctos cinereus*): insights into koala retrovirus transcription and sequence diversity. BMC Genomics. 2014;15:786.

16. Morris KM, O'Meally D, Zaw T, Song X, Gillett A, Molloy MP, et al. Characterisation of the immune compounds in koala milk using a combined transcriptomic and proteomic approach. Scientific Reports. 2016;6:e35011.

17. Belov K, Sanderson CE, Deakin JE, Wong ESW, Assange D, McColl KA, et al. Characterization of the opossum immune genome provides insights into the evolution of the mammalian immune system. Genome Research. 2007;17:982-91.

18. Cho H-s, Yum J, Larivière A, Lévêque N, Le QVC, Ahn B, et al. Opossum Cathelicidins Exhibit Antimicrobial Activity Against a Broad Spectrum of Pathogens Including West Nile Virus. Frontiers in Immunology. 2020;11(347).

19. Peel E, Cheng Y, Djordjevic JT, Fox S, Sorrell TC, Belov K. Cathelicidins in the Tasmanian devil (*Sarcophilus harrisii*). Scientific Reports. 2016;6:e35019.

20. Peel E, Cheng Y, Djordjevic JT, Kuhn M, Sorrell T, Belov K. Marsupial and monotreme cathelicidins display antimicrobial activity, including against methicillin-resistant *Staphylococcus aureus*. Microbiology. 2017;163:1457-65.

21. Peel E, Cheng Y, Djordjevic JT, O’Meally D, Thomas M, Kuhn M, et al. Koala cathelicidin PhciCath5 has antimicrobial activity, including against Chlamydia pecorum. PLOS ONE. 2021;16(4):e0249658.

22. Zhou Y, Shearwin-Whyatt L, Li J, Song Z, Hayakawa T, Stevens D, et al. Platypus and echidna genomes reveal mammalian biology and evolution. Nature. 2021.
